# Organophosphate Detoxification and Acetylcholinesterase Reactivation Triggered by Zeolitic Imidazolate Framework Structural Degradation

**DOI:** 10.1021/acsami.3c18855

**Published:** 2024-02-12

**Authors:** Javier D. Martin-Romera, Emilio Borrego-Marin, Pedro J. Jabalera-Ortiz, Francesco Carraro, Paolo Falcaro, Elisa Barea, Francisco J. Carmona, Jorge A. R. Navarro

**Affiliations:** † Departamento de Química Inorgánica, Universidad de Granada, Av. Fuentenueva S/N, Granada 18071, Spain; ‡ Institute of Physical and Theoretical Chemistry, TU Graz, Stremayrgasse 9, Graz A-8010, Austria

**Keywords:** controlled drug delivery, nerve agent, metal−organic framework, hydrolysis, catalysis

## Abstract

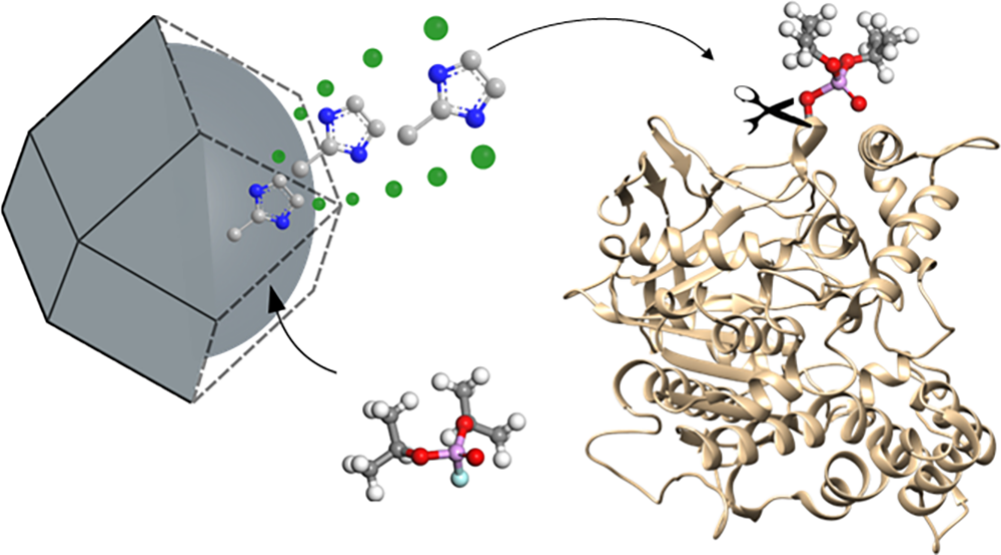

Organophosphate (OP) toxicity is related to inhibition of acetylcholinesterase (AChE) activity, which plays a key role in the neurotransmission process. In this work, we report the ability of different zinc zeolitic imidazolate frameworks (ZIFs) to behave as potential antidotes against OP poisoning. The Zn–L coordination bond (L = purine, benzimidazole, imidazole, or 2-methylimidazole) is sensitive to the G-type nerve agent model compounds diisopropylfluorophosphate (DIFP) and diisopropylchlorophosphate, leading to P–X (X = F or Cl) bond breakdown into nontoxic diisopropylphosphate. P–X hydrolysis is accompanied by ZIF structural degradation (Zn–imidazolate bond hydrolysis), with the concomitant release of the imidazolate linkers and zinc ions representing up to 95% of ZIF particle dissolution. The delivered imidazolate nucleophilic attack on the OP@AChE adduct gives rise to the recovery of AChE enzymatic function. P–X bond breakdown, ZIF structural degradation, and AChE reactivation are dependent on imidazolate linker nucleophilicity, framework topology, and particle size. The best performance is obtained for 20 nm nanoparticles (NPs) of Zn(2-methylimidazolate)_2_ (sod ZIF-8) exhibiting a DIFP degradation half-life of 2.6 min and full recovery of AChE activity within 1 h. 20 nm sod ZIF-8 NPs are not neurotoxic, as proven by in vitro neuroblastoma cell culture viability tests.

## Introduction

Organophosphate (OP)-based pesticides and chemical warfare nerve agents are extremely toxic compounds, as a consequence of the central nervous system damage by irreversible inhibition of acetylcholinesterase (AChE) activity.^1,2^ In this regard, much effort has been devoted to develop materials for protection against exposure to OP compounds, particularly filters and detoxication catalysts.^3−7^ However, disruptive antidotes for OP poisoning are underexplored.^8,9^ In this regard, current OP poisoning treatment is based on the nucleophilic attack of oxime-based drugs on the phosphorylated AChE (OP@AChE) adduct with the aim to restore AChE function and recover a normal neurotransmission process. However, while low oxime concentrations lead to an inefficient AChE reactivation, large amounts of reactivators have shown to inhibit the enzyme and lead to severe toxicity issues.^10,11^ In addition, poor permeability through the blood–brain barrier limits the efficiency of the oxime treatment.^12^

**Scheme 1:**
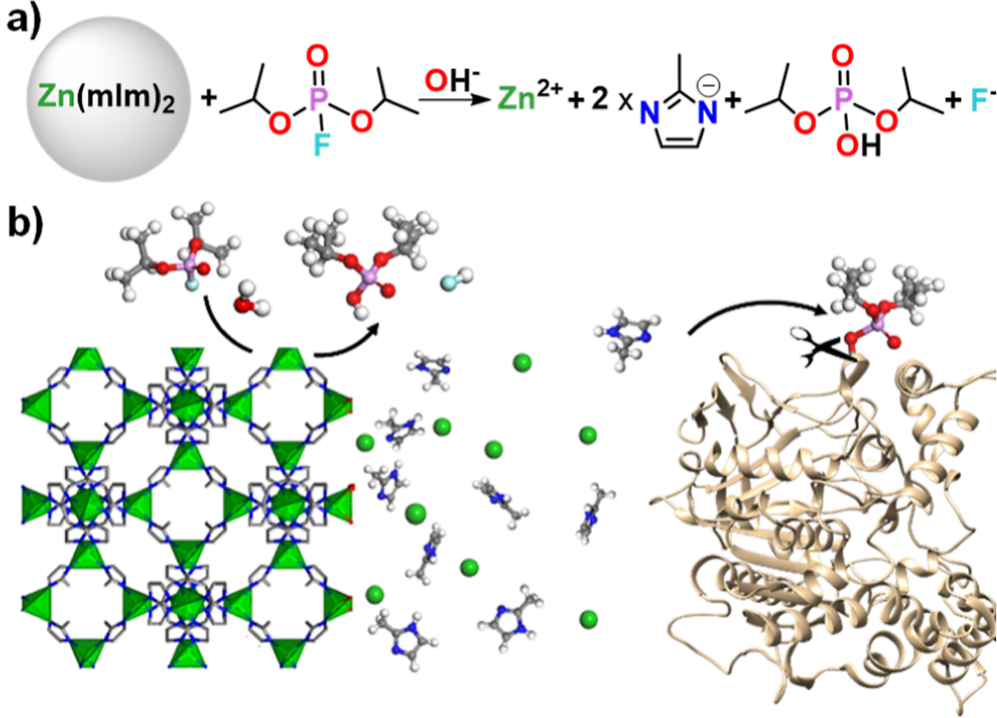
(a) G-Type Nerve Agent Simulant Diisopropylfluorophosphate (DIFP) Hydrolytic Degradation into Nontoxic Diisopropylphosphate (DIP) Triggered by Structural Degradation of the Zeolitic Imidazolate Framework (ZIF) Crystal Surface; (b) Schematic Representation of Nucleophilic Attack of Imidazolate, Released after ZIF Structural Degradation, to OP-Inhibited AChE; Atom Color Code in (b): C (Gray), F (Cyan), H (Pale Grey), N (Blue), O (Red), P (Magenta), and Zn (Green Tetrahedra)

Consequently, there is a need to develop disruptive materials that can mitigate OP poisoning. Here we propose that ZIFs, a subclass of metal–organic frameworks (MOFs) with a rich structural diversity, amenable chemistry, particle size control,^13−16^ and biocompatibility,^17−19^ might behave as antidotes of OP poisoning. Our starting hypothesis is that the nucleophilic nature and low toxicity of imidazolate linkers [intraperitoneal mouse LD_50_ of 800, 445, 300, and 480 mg kg^–1^ for purine (pIm), benzimidazole (bIm), imidazole (Im), and 2-methylimidazole (mIm), respectively],^20^ commonly used for ZIF synthesis, might behave as reactivators of OP@AChE (Scheme 1). In this regard, the zinc–imidazolate coordination bond has been shown to be highly sensitive to biological media, resulting in the controlled structural degradation of ZIFs and the concomitant release of its constituents (zinc ions and imidazolate linkers) and cargo (bioactive molecules and biomolecules).^21^ It is noteworthy that the structural degradation is advantageous since it prevents the accumulation of nanoparticles (NPs) in the body.^22^ Indeed, in vivo biosafety of ZIF-8 NPs and degradation products thereof has been recently demonstrated.^23^

In addition, zinc-azolate MOFs (including some examples of ZIFs) have been shown to exhibit catalytic activity toward the degradation of organophosphorus compounds, mimicking the active site of phosphohydrolase enzymes as a consequence of a suitable balance of Zn Lewis acidity and linker basicity.^24−27^ However, all previous reports require strong basic buffered media (pH 9–11) to exhibit hydrolase-like activity, which is far from physiological conditions (pH 7.4).

In this work, we have explored the different physicochemical characteristics of zinc-ZIFs governing their OP-triggered structural degradation and reactivation of the phosphorylated AChE adduct by released imidazolate linkers under simulated physiological conditions (Scheme 1). Specifically, we have studied the effect of different physicochemical features of ZIFs as OP antidotes, namely, (i) nucleophilicity of imidazolate linkers; (ii) framework topologies; and (iii) particle sizes.

## Experimental Section

### Materials and Reagents

All chemicals and solvents were commercially available from commercial sources and used without further purification. ZIF materials and Zn(pymo)_2_ (pymo = pyrimidin-2-olate) were synthesized as previously reported in the literature.^22,28−33^

### Physical and Chemical Methods of Characterization

Powder X-ray diffraction (PXRD) data were obtained at room temperature on a Bruker D2 PHASER diffractometer using Cu-Kα radiation (λ = 1.5418 Å), collecting in the 5–35° 2θ range, with steps of 0.02° and a time per step of 0.5 s. The samples were deposited in the hollow of a zero-background silicon sample holder. Elemental analysis (EA) was performed in an elemental analyzer Thermo Scientific FLASH 2000. ^1^H and ^31^P nuclear magnetic resonance spectroscopy (^1^H- and ^31^P NMR) data were obtained with a Bruker Nanobay AVANCE III HD 400 MHz (2-channel) NMR spectrometer. Thermogravimetric analysis (TGA) was carried out by a thermogravimetric analyzer Shimadzu mod. TGA-50H. Nitrogen adsorption isotherms at 77 K were measured on a Micromeritics 3Flex adsorption analyzer. Fourier transform infrared (FTIR) measurements were obtained with a Bruker spectrophotometer. Scanning electron microscopy (SEM) images were obtained on a Carl Zeiss SMT Auriga. Transmission electron microscopy (TEM) images were obtained on a FEI Talos F200X microscope. Microwave synthesis was carried out using an Anton Paar Monowave 300 reactor. DIFP adsorption experiments were performed in an Agilent 8860 GC chromatograph with an FID detector. The column used was HP-5 of 50 m length, 0.320 mm diameter, and 1.05 μm thickness. Enzymatic assays were performed in a Tecan Infinite 200 PRO NanoQuant.

### DIFP and Diisopropylchlorophosphate Degradation Studies

#### Gas Chromatography Studies

Hydrolytic degradation of DIFP by different ZIFs was assessed by gas chromatography analysis. In a typical experiment, ZIF-20 (0.084 mmol), ZIF-11 (0.084 mmol), Zn(Im)_2_ (0.084 mmol), sod ZIF-8_2 μm (0.084 mmol), sod ZIF-8_1.2 μm (0.084 mmol), sod ZIF-8_240 nm (0.084 mmol), sod ZIF-8_20 nm (0.084 mmol), ZIF-L (0.084 mmol), or ZIF-EC-1 (0.029 mmol) was mixed with DIFP (2.5 μL, 0.015 mmol) and dimethylformamide (DMF, 1.08 μL, 0.015 mmol, internal reference) in 0.5 mL of Tris–HCl (0.1 M, pH 7.4) and stirred at room temperature. The evolution of the DIFP concentration was monitored by gas chromatography. Hydrolytic degradation of diisopropylchlorophosphate (DICP) by sod ZIF-8 of different particle size (20 nm–2 μm) was monitored by gas chromatography analysis following a similar protocol using deuterated Tris–DCl.

#### ^1^H and ^31^P NMR Spectroscopy Studies

Hydrolytic degradation of DIFP by different ZIFs was studied by ^1^H and ^31^P NMR. In a typical experiment, Zn(Im)_2_ (0.084 mmol), sod ZIF-8_2 μm (0.084 mmol), sod ZIF-8_1.2 μm (0.084 mmol), sod ZIF-8_240 nm (0.084 mmol), sod ZIF-8_20 nm (0.084 mmol), ZIF-L (0.084 mmol), or ZIF-EC-1 (0.029 mmol) was mixed with DIFP (0.029 M) and dimethylphosphate (0.029 M, internal reference) in deuterated Tris–HCl (0.1 M, pH 7.4, 0.5 mL). At different times, the mixtures were analyzed by ^1^H and ^31^P NMR to quantify the concentration of DIFP and imidazolate linkers (mIm or Im) in the solution. Hydrolytic degradation of DICP by sod ZIF-8 of different particle sizes (20 nm–2 μm) was monitored by ^1^H and ^31^P NMR using a similar protocol.

As control experiments, the ability of Zn^2+^ ions [Zn(NO_3_)_2_·6H_2_O, 0.100 mmol] and mIm (0.020 mmol) to hydrolyze DIFP in deuterated Tris–HCl (0.1 M, pH 7.4, 0.5 mL) and dimethylphosphate (0.029 M, internal reference) after 24 h was also evaluated by ^1^H NMR.

### Enzymatic Assays

In all experiments, the AChE activity was assessed by using a colorimetric method which employs indoxyl acetate as a substrate that is converted into indigo blue.^34^ The concentration of the enzymatic product was calculated by UV–vis spectroscopy at λ = 620 nm, and these data were used to calculate the enzymatic activity (ϵ = 22,140 M^–1^ cm^–1^).^35^ We selected this method to evaluate AChE activity, instead of the classical Ellman method, as a consequence of acetylthiocholine’s sensitivity to nucleophiles (i.e., oximes) which leads to false positives.^36^

#### AChE Reactivation Assays

First, the AChE reactivation activity of the different imidazolate linkers (pIm, Im, and mIm) toward 50% inhibited AChE was assessed. The low solubility of bIm in aqueous solution prevents the performance of the AChE reactivation assay with this imidazolate linker. In a typical experiment, 24-well culture plates were filled with 725 μL of Tris–HCl (0.1 M, pH 7.4), 25 μL of an AChE aqueous solution (75 U/mL), 100 μL of an aqueous DIFP solution ([DIFP] final concentration of 5.0 × 10^–6^ M corresponding to 50% inhibition of AChE enzymatic activity), and 100 μL of an aqueous solution of each imidazolate linker at different concentrations (final concentration: 10^–4^ to 0.1 M). The mixtures were then incubated for 1 h at 37 °C. Afterward, 50 μL of indoxyl acetate solution in isopropanol (3.0 mM) was added, and the mixtures were incubated for an additional 30 min. Finally, 3.33 mL of DMSO was added to solubilize the enzymatic product (indigo blue) and the absorbance of the solutions at λ = 620 nm was collected. Additional experiments were carried out to estimate the enzymatic activity of uninhibited AChE and 50% inhibited AChE with DIFP ([DIFP] final concentration of 5.0 × 10^–6^ M). For the uninhibited AChE assay, the procedure was the same as for the reactivation assays except that DIFP and imidazolate aqueous solutions were replaced by 200 μL of water in the mixture. For the 50% inhibited AChE assay, the procedure was the same as for the reactivation assays except that 100 μL of water was added to the mixture instead of 100 μL of aqueous imidazolate solution.

In a second step, the AChE reactivation activity of the imidazolate linker released after ZIF degradation in a simulated biological fluid was also assessed. In a typical experiment, Zn(Im)_2_ (0.588 mmol), sod ZIF-8_2 μm (0.588 mmol), sod ZIF-8_20 nm (0.588 mmol), ZIF-L (0.588 mmol), or ZIF-EC-1 (0.203 mmol) was suspended in 3.5 mL of Tris–HCl (0.1 M, pH 7.4) for 24 h. Afterward, the supernatant was separated from the solid by centrifugation (15,000 rpm × 5 min). Then, 24-well culture plates were filled with 825 μL of supernatant, 25 μL of an AChE aqueous solution (75 U/mL), and 100 μL an aqueous DIFP solution (final concentration of 5.0 × 10^–6^ M). The mixtures were then incubated for 1 h at 37 °C. Afterward, 50 μL of indoxyl acetate solution in isopropanol (3.0 mM) was added and the mixtures were incubated for an additional 30 min. Finally, 3.33 mL of DMSO was added to solubilize the enzymatic product (indigo blue) and the absorbance of the solutions at λ = 620 nm was collected. Additional experiments were carried out to estimate the enzymatic activity of uninhibited AChE and inhibited AChE with DIFP following the same procedure as above.

#### Detoxification Studies

Additional enzymatic assays, termed detoxification, were carried out to determine if ZIFs were able to mitigate the inhibitory effect of DIFP on AChE activity after different times of incubation (1, 3, and 24 h).

In this case, sod ZIF-8_2 μm (0.084 mmol), sod ZIF-8_20 nm (0.084 mmol), ZIF-L (0.084 mmol), or ZIF-EC-1 (0.029 mmol) was mixed with DIFP (0.029 M) in 0.5 mL of Tris–HCl (0.1 M, pH 7.4) and stirred at room temperature. After different time frames (1, 3, and 24 h), the supernatant was separated from the solid by centrifugation (15,000 rpm × 5 min) and diluted 580 times. Then, a 24-well culture plate was filled with 825 μL of Tris–HCl (0.1 M, pH 7.4), 25 μL of an aqueous solution of AChE (75 U/mL), and 100 μL of the diluted supernatant. The mixtures were then incubated for 1 h at 37 °C. Afterward, 50 μL of indoxyl acetate solution in isopropanol (3.0 mM) was added and the mixtures were incubated for an additional 30 min. Finally, 3.33 mL of DMSO was added to solubilize the enzymatic product (indigo blue) and the absorbance of the solutions at λ = 620 nm was collected. Additional experiments were carried out to estimate the enzymatic activity of uninhibited AChE and inhibited AChE with DIFP, following the same protocol as in the reactivation assays.

### Cell Culture and Viability Assays

The SH-SY5Y human neuroblastoma cell culture, obtained from the Centre of Scientific Instrumentation of the University of Granada, was cultivated as a suspension in RPMI-1640 medium (Sigma-Aldrich R5886) supplemented with 20% FBS (Sigma-Aldrich F4135), 4 mM l-glutamine (Sigma-Aldrich G7513), 1 mM sodium pyruvate (Sigma-Aldrich S8636), and 4.5 mg/mL glucose. Cells were incubated at 37 °C in a humidified atmosphere with 5% CO_2_ and maintained using standard cell culture techniques. Cytotoxicity of the sod ZIF-8_240 nm and sod ZIF-8_20 nm was determined by the colorimetric MTS assay.

## Results and Discussion

### ZIF Materials Selection and Synthesis

**Figure 1:**
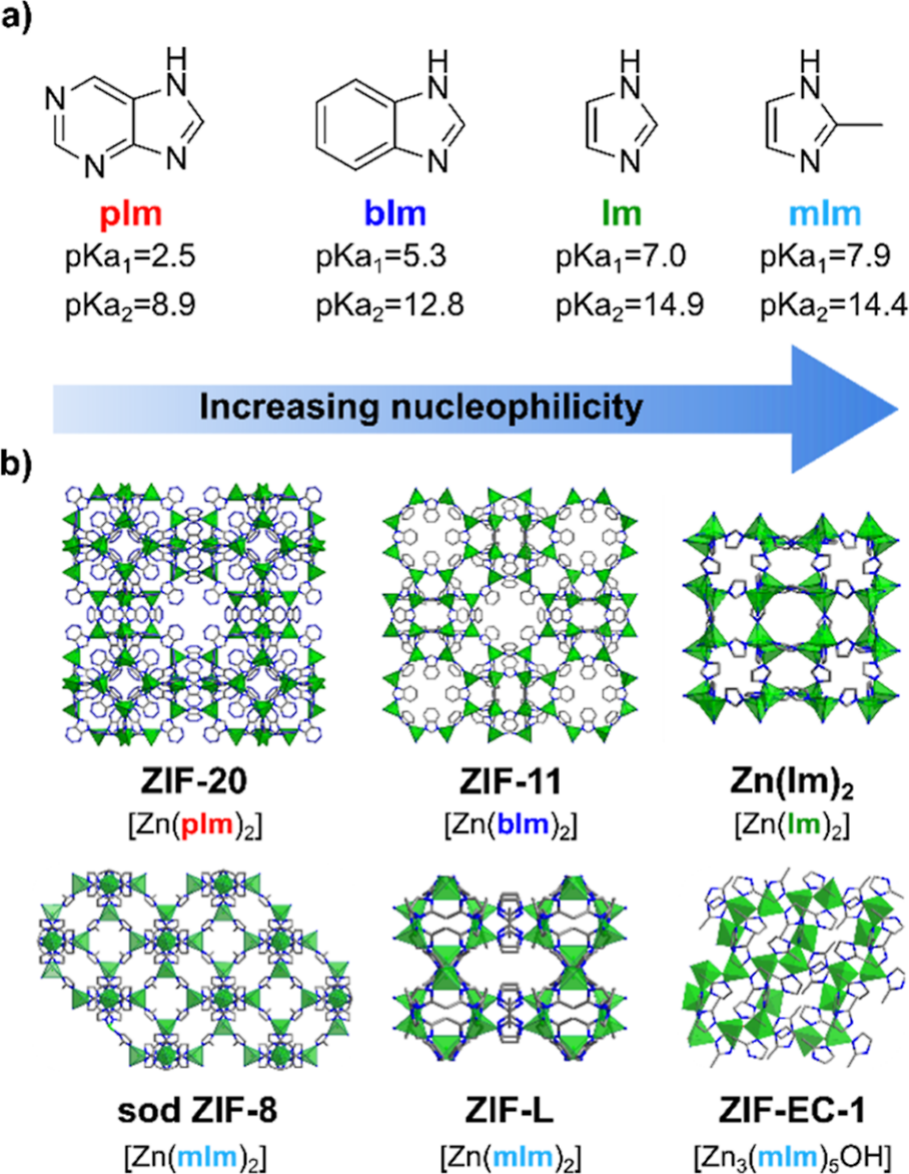
(a) Selected imidazolate linkers of increasing basicity/nucleophilicity. (b) Structures of prepared ZIFs Zn(pIm)_2_ (ZIF-20, porous zeolite A), Zn(bIm)_2_ (ZIF-11, porous zeolite RHO), Zn(Im)_2_ (3D nonporous), ZIF-L (layered), sod ZIF-8 (3D, sodalite), and ZIF-EC-1 (3D, nonporous). Atom color code in (b): C (gray), N (blue), O (red), and Zn (green tetrahedra).

To probe the above hypothesis, we have selected a series of imidazolate linkers with increasing basicity (nucleophilicity), namely, purine (pIm), benzimidazole (bIm), imidazole (Im), and 2-methylimidazole (mIm) (Figure 1a) to prepare six distinct Zn-based imidazolate frameworks, namely, Zn(pIm)_2_ (ZIF-20, porous zeolite A),^37^ Zn(bIm)_2_ (ZIF-11, porous zeolite RHO),^13^ Zn(Im)_2_ (3D nonporous),^29^ Zn(mIm)_2_ (ZIF-L, layered, nonporous to N_2_),^31,38^ Zn(mIm)_2_ (sod ZIF-8, 3D, porous sodalite),^13^ and Zn_3_(mIm)_5_(OH) (ZIF-EC-1, 3D, nonporous to N_2_)^32,39^ (Figure 1b). All ZIFs have the same formula [Zn(imidazolate)_2_] but different topology except for ZIF-EC-1, which can be considered a defective ZIF structure in which one-sixth of the imidazolate linkers have been replaced by a hydroxide bridge. These systems were prepared according to literature methods, in the form of micrometer particles, and their chemical, structural, and morphological properties were confirmed by PXRD, N_2_ adsorption isotherms, TGA, EA, infrared spectroscopy (FTIR), and SEM (Figures S1, S3–S6 and Table S1).

### Impact of Linker Basicity and ZIF Topology on DIFP Hydrolysis

**Figure 2:**
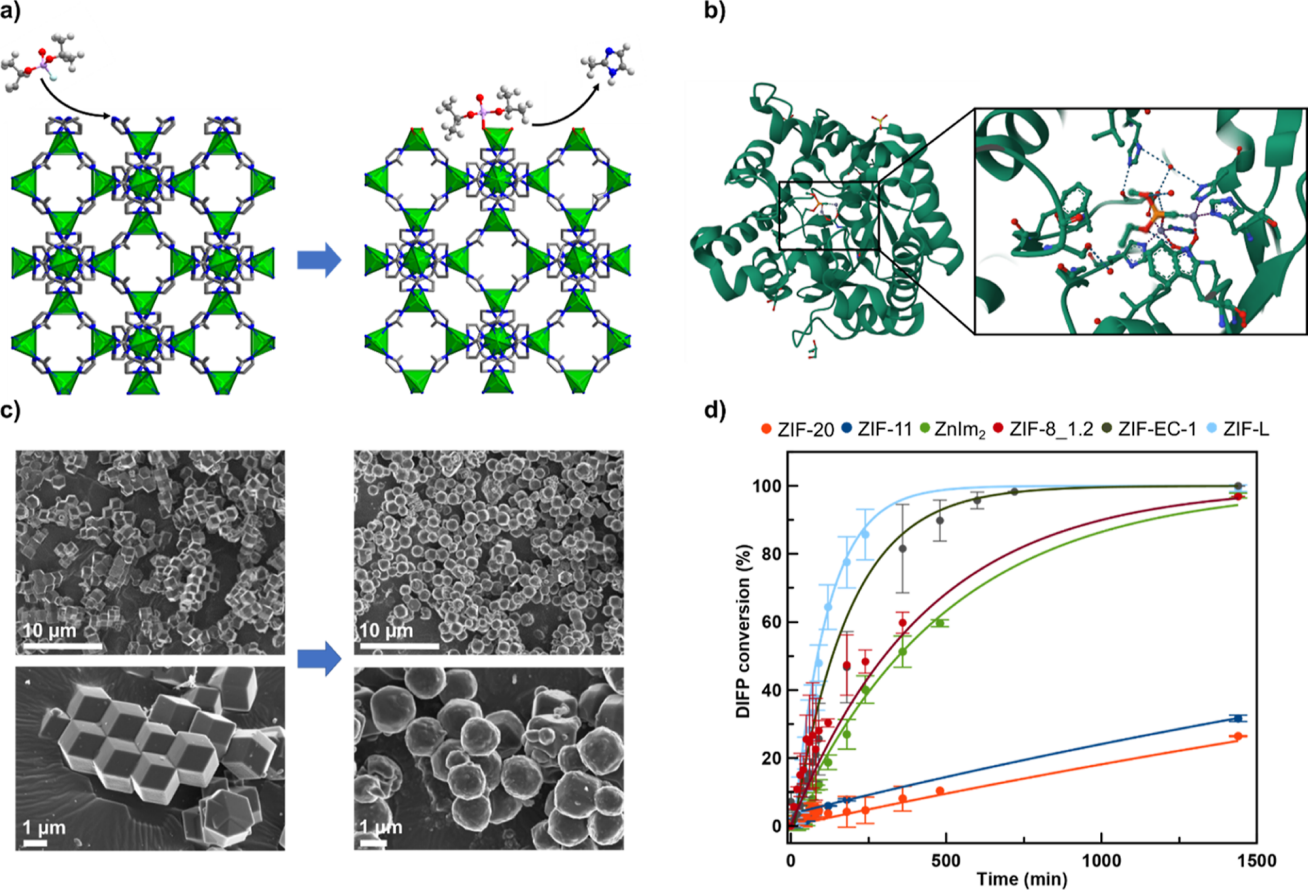
ZIFs reactivity toward model nerve agent DIFP: (a) ZIF structural degradation reaction triggered by DIFP interaction with the 1 0 0 crystal face of sod ZIF-8. (b) Structure of the organophosphate@phosphotriesterase adduct highlighting the structural details of the catalytic center.^40^ (c) SEM images of sod ZIF-8 particles before and after 24 h exposure to DIFP (0.17 M, 0.5 mL) in Tris–HCl (0.1 M, pH 7.4). (d) Profiles of DIFP (0.029 M, 0.5 mL) hydrolytic degradation, under simulated biological conditions (Tris–HCl, 0.1 M, pH 7.4), showing enhanced catalytic activity of ZIFs (0.084 mmol) upon an imidazolate linker’s basicity increase. Atom color code in (a): C (gray), F (cyan), H (pale gray), N (blue), O (red), P (magenta), and Zn (green tetrahedra). Color code in (b) ZIF-20 (red), ZIF-11 (blue), Zn(Im)_2_ (green), sod ZIF-8_1.2 μm (granate), ZIF-EC-1 (gray), and ZIF-L (cyan).

Once we proved the chemical and crystal-phase purity of the selected ZIFs, we explored the impact of linker basicity and framework topology on nerve agent hydrolytic degradation. In the first step, we have studied the reactivity of the as-synthesized ZIFs (0.084 mmol, micrometer particle size) toward the model nerve agent DIFP (0.029 M), under simulated biological conditions (Tris–HCl, 0.1 M, pH 7.4) (Figure 2). The results are indicative that only ZIFs based on the two most basic linkers of the series, Im and mIm, lead to significant P–F bond hydrolysis of DIFP, yielding nontoxic DIP (Figures 2a,d, S7a, S8 and S10–S14). The half-life times *t*_1/2_ for DIFP degradation follow the trend 75 min (ZIF-L) < 124 min (ZIF-EC-1) < 238 min (sod ZIF-8_1.2 μm) < 333 min [Zn(Im)_2_] (Figures 2d and S8). By contrast, ZIF-20 and ZIF-11 systems based on pIm and bIm linkers, of lower basicity, exhibit a poorer hydrolytic activity, with DIFP degradation reaching ∼25% after 24 h. These results agree with an important imidazolate linker tuning of acid–base properties of the catalytically active Zn-(μ-Im)-Zn sites at the ZIF crystal surface. The divergence on catalytic activity for ZIF-L, ZIF-EC-1, and sod ZIF-8 microparticles should be related to framework topology/crystalline phase and crystal size differences (Figures 1 and S6). It is noteworthy that DIFP reaction with ZIFs’ crystal surface triggers their structural degradation with imidazolate linker and Zn^2+^ release (Figure 2a) as proven by ^1^H NMR (Figures S10–S13 and Table S3). The imidazolate release for the pIm-, bIm-, and Im-based ZIFs is 1 order of magnitude lower than the mIm-based ZIF systems, pointing to a clear correlation between structural degradation and DIFP hydrolytic breakdown. SEM images confirm DIFP-induced particle surface etching for mIm-based ZIFs (Figures 2c and S23–S26). Under the essayed conditions ([DIFP] = 0.18 M, Tris–HCl, 0.1 M, pH 7.4), ZIF-L, ZIF-EC1, and sod ZIF-8 particle degradation is in the order of 30–35% (Figures S23–S26).^41^ Control experiments in the absence of the nerve agent model compound lead to lower ZIF structural degradation (Tables S3 and S5) and no apparent crystal morphology modification (Figures S23–S25). Moreover, additional control experiments with either Zn(NO_3_)_2_ or imidazolate linkers give rise to a poor DIFP hydrolytic degradation (Figure S17), pointing to a synergistic interplay between Lewis acidity of Zn^2+^ and basicity of imidazolate and/or hydroxide residues on the essayed ZIF crystals’ surface, resembling the Zn_2_(μ–OH)(imidazole)_4_ phosphotriesterase catalytic center (Figure 2b).^42^

To further support our claim, we have carried out a similar study with a related pyrimidine-based MOF Zn(2-pymo)_2_ (2-Hpymo: 1*H*-pyrimidin-2-one).^33^ The results are indicative of both poor catalytic activity (∼10% DIFP hydrolysis after 24 h) and negligible framework structural degradation (see Supporting Information). This behavior can be related to the lower basicity of 2-Hpymo (p*K*_a_1__: 2.24; p*K*_a_2__: 9.17) in comparison to that of imidazole linkers.

### Particle Size Effect on Hydrolytic Degradation of G-Type Nerve Agent Analogues

**Figure 3:**
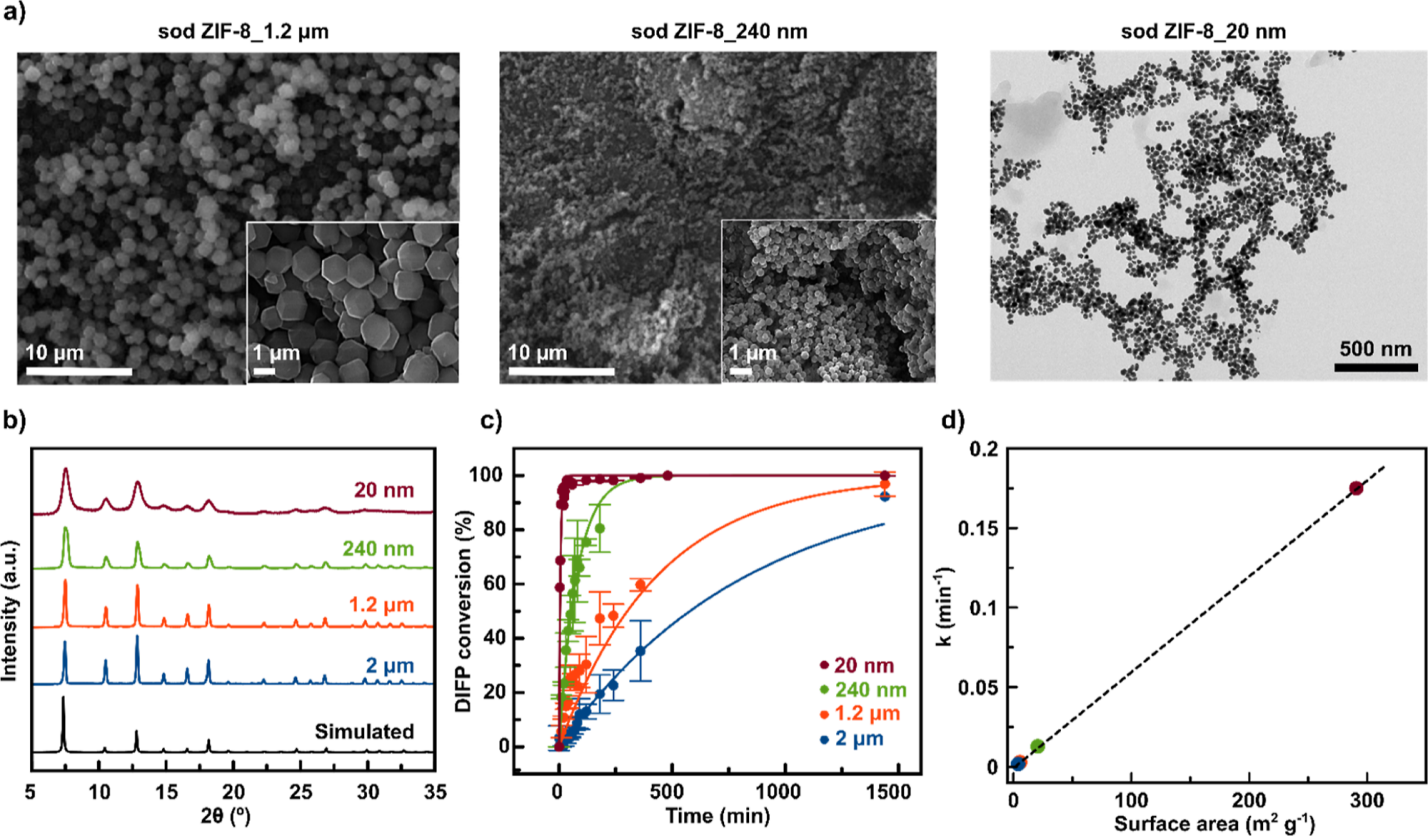
Sod ZIF-8 size-dependent catalytic activity: (a) SEM and TEM images of sod ZIF-8 with decreasing particle size; (b) XRPD patterns for sod ZIF-8 particles with decreasing sizes; (c) sod ZIF-8 particle size-dependent DIFP (0.029 M, 0.5 mL) hydrolytic degradation, under simulated biological conditions (Tris–HCl, 0.1 M, pH 7.4); and (d) correlation between the geometrical external surface^43^ of sod ZIF-8 particles and kinetics constant values for DIFP degradation.

After probing the important role of mIm basicity, we focused on the impact of ZIF particle size on degradation of G-type nerve agent surrogates DIFP and DICP.^44,45^ With this aim, we have taken advantage of the crystal-phase stability and amenable size tuning formation of sodalite ZIF-8 crystals. By contrast, the metastable nature of ZIF-L does not allow pure phase particle downsizing.^38^ The synthesis of sod ZIF-8 NPs was carried out according to literature methods by variation of the zinc salt-to-linker ratio and/or addition of *n*-butylamine as a modulating agent.^30^ In this way, ZIF-8 sod particles ranging from 20 nm to 2 μm have been prepared (Figures 2c and 3a). XRPD patterns (including Le Bail analysis),^46^ EA, TGA, FTIR, and N_2_ adsorption characterization (Figures 3b, S1–S5 and Tables S1 and S2) confirm the formation of chemically pure and crystal-phase pure sod ZIF-8 particles. The particle size of the different materials was estimated based on electron microcopy imaging (SEM and TEM) (Figures 3a and S6). It is noteworthy that there is good agreement between the estimated NP size from TEM images (20–23 nm) and crystal-phase domain (24 nm) calculated from Lorentzian convolution of Bragg peaks for the smallest sod ZIF-8 NPs (Figures 3a, S29 and Table S2).

Sod ZIF-8 particles (0.084 mmol) were exposed to the model nerve agent DIFP (0.029 M, 0.5 mL) in simulated biological media (Tris–HCl, 0.1 M, pH 7.4). The dependence of half-life times *t*_1/2_ on NP size for DIFP degradation follows the trend 2.6 min (20 nm) < 43 min (240 nm) < 249 min (1.2 μm) < 578 min (2 μm) (Figures 3c and S8). Sod ZIF-8 particles exhibit a similar catalytic activity trend toward P–Cl bond hydrolysis, with 20 nm sod ZIF-8 NPs quantitatively degrading DICP to DIP in less than 1 min (Figures S6b and S17–S21) and the concomitant release of the mIm linker (Table S3). It is noteworthy that there is a clear correlation between the calculated external surface of sod ZIF-8 particles and DIFP catalytic degradation kinetics’ constant values (Figure 3d).^43^ These results suggest that nerve agent mimic breakdown is taking place at the particle external surface. Indeed, SEM and TEM images of sod ZIF-8 NPs exposed to DIFP ([DIFP] = 0.18 M, pH 7.4) indicate a significant surface etching which increases upon particle downsizing (volume loss of 31% for 2 μm particles, 47% for 240 nm particles, and 95% for 20 nm particles) (Figures S25–S28). This tendency is also reflected on the higher extension of linker release with [mIm] reaching 0.05 M for nanometer-sized particles (Table S2). The high extent of 20 nm NPs’ structural degradation upon exposure to DIFP down to ultrasmall 7 nm NPs should be considered beneficial to avoid NP bioaccumulation facilitating their body clearance.^23^ The size dependence of ZIF structural degradation is also reflected in the high linker release with [mIm] reaching 0.05 M for the nanometer-sized particles (Table S3).

### ZIF Nerve Agent Detoxification: Dual P–F Bond Breakdown and AChE Reactivation

**Figure 4:**
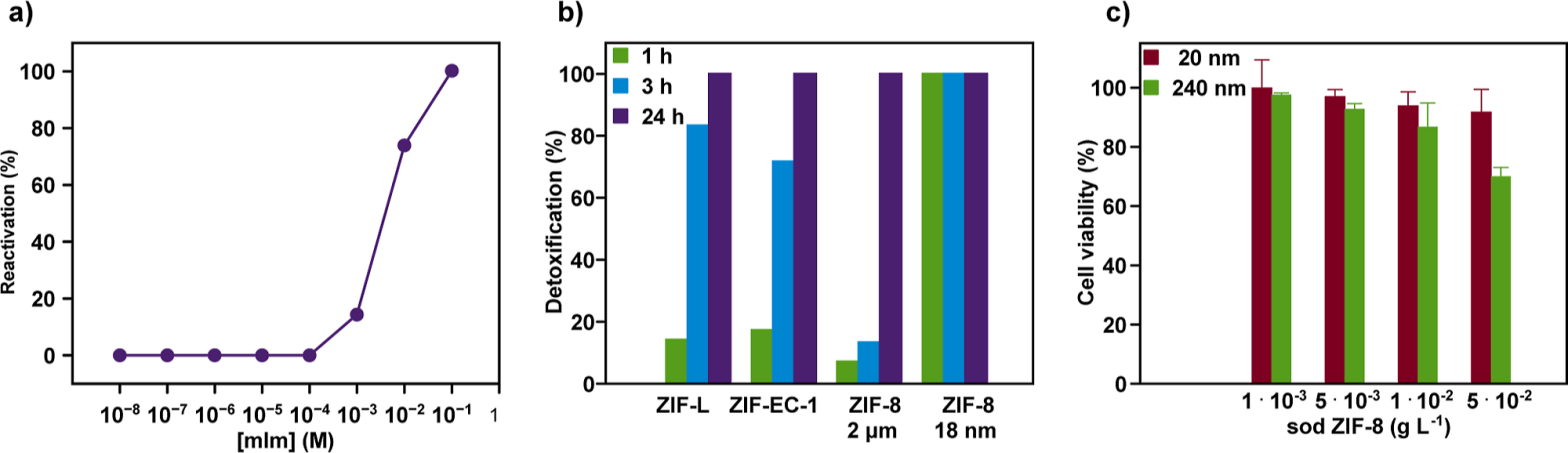
(a) Reactivation profile of DIFP inhibiting AChE (50% activity in comparison to free AchE) by mIm (Tris–HCl, 0.1 M, pH 7.4). (b) Detoxification effect of ZIF-L, ZIF-EC-1, sod-ZIF-8 microparticles (1–2 μm), and sod ZIF-8 NPs (20 nm) after 1, 3, and 24 h of exposition to DIFP on AChE activity evaluated at simulated biological conditions (Tris–HCl, 0.1 M, pH 7.4). (c) In vitro cytotoxicity evaluation of 20 and 240 nm sod ZIF-8 NPs on human neuroblastoma model neurons.

We have evaluated whether the DIFP-induced ZIF structural degradation can recover the enzymatic activity of the OP-inhibited AChE (OP@AChE). In the first step, incubation of AChE with DIFP (5 × 10^–6^ M) leads to 50% enzymatic activity inhibition. In the second step, we evaluated the capacity of the different imidazolate linkers to reactivate the DIFP@AChE system. The results show that the mIm linker behaves as an AChE reactivator at [mIm] above 1 × 10^–4^ M under simulated biological conditions (Tris–HCl, 0.1 M, pH 7.4) (Figure 4a). By contrast, the rest of imidazole linkers exhibit a moderate 25% reactivation activity at the highest concentration (0.1 M) which is related to their lower basicity/nucleophilicity (Figure S30). Similarly, the 2-Hpymo linker exhibits a very low AChE reactivation of 4% at 0.1 M (Figure S30) which is in line with its lower nucleophilicity.

In view of this screening, we have studied the ability of mIm released from ZIF structural degradation to reactivate OP-inhibited AChE. Specifically, supernatants derived from 24 h incubation of ZIF-L, sod ZIF-8, ZIF-EC-1 microparticles, and sod ZIF-8 NPs (20 nm) under simulated biological conditions ([mIm] = 0.6–2.0 × 10^–3^ M, Tris–HCl, 0.1 M, pH 7.4) were evaluated. The results show that these supernatants can recover 10–54% of the AChE enzymatic activity (Table S5). As expected, the most active supernatant is the 20 nm sod ZIF-8 NPs which can recover 54% of the enzymatic activity.

Next, we evaluated the dual benefit of DIFP degradation at the ZIF crystal surface and AChE reactivation by the structurally degraded mIm-based ZIF materials. Specifically, ZIF-L, sod ZIF-8, ZIF-EC-1 microparticles, and 20 nm sod ZIF-8 NPs (0.084 mmol) were dispersed in a DIFP solution (0.029 M, 0.5 mL) under simulated biological conditions (Tris–HCl, 0.1 M, pH 7.4) and the mixture was kept at 37 °C for 1, 3, and 24 h incubation times (see Supporting Information). Afterward, the supernatants collected from ZIF suspensions were evaluated for the mitigation of the inhibitory effect of DIFP on AChE activity. We term this process as detoxification (Table S6). The detoxification profiles exerted by ZIFs (Figure 4b) follow a similar trend to DIFP hydrolytic degradation (Figures 2d, 3c and S7a). Indeed, the most catalytically active 20 nm sod ZIF-8 NPs can recover 100% of the AChE activity within 1 h (Figure 4b). By contrast, ZIF-L, ZIF-EC-1, and sod ZIF-8 microparticles exhibit a poorer detoxification at 1 h, increasing to 83, 72, and 17%, enzymatic activity recovery after 3 h, respectively. This trend is in line with the respective catalytic activities (see above). All materials suppress the inhibitory effect of DIFP on AChE function after 24 h of incubation (Figure 4b). These results agree on the benefit of nerve agent detoxification of DIFP-induced ZIF structural degradation resulting in P–F bond hydrolysis coupled to AChE reactivation. Finally, we have evaluated the in vitro neurotoxicity of the 20 and 240 nm sod ZIF-8 NPs toward the SH-SY5Y human neuroblastoma cell culture.^47^ The results shown in Figure 4c are indicative that cell viability is poorly affected by sod ZIF-8 NPs of 20 nm exhibiting a negligible in vitro neurotoxicity while 240 nm sod ZIF-8 NPs exhibit a small degree of toxicity (70% cell viability) at the largest assayed concentration (0.05 g L^–1^).

## Conclusions

In summary, we have demonstrated that the sensitivity of the Zn–imidazolate coordination bond to G-type nerve agent model compounds leads to P–X hydrolysis, into nontoxic DIP, and triggers framework structural degradation at the ZIF crystal surface, with concomitant release of the corresponding imidazolate linker. P–X bond hydrolysis and ZIF crystal etching are highly interconnected and are dependent on linker basicity, framework topology, and crystal size. In this regard, 20 nm sod ZIF-8 NPs are highly efficient in both P–X bond breakdown and release of mIm, from their structural degradation, that can in turn nucleophilically attack the OP-AChE adduct. These two processes result in the recovery of the original AChE enzymatic function, thereby reversing OP poisoning. The cascade reaction reported here is highly advantageous since it is a single material with a dual function. Finally, we demonstrate that 20 nm sod ZIF-8 NPs exhibit a low in vitro neurotoxicity.

## Supporting Information Available

Synthetic protocols, full characterization of ZIF materials, details of DIFP detoxification, and AChE inhibition and reactivation assays (PDF)
