# Supplementary material for: Organophosphate Detoxification and Acetylcholinesterase Reactivation Triggered by Zeolitic Imidazolate Framework Structural Degradation
Source: ACS Appl Mater Interfaces. 2024 Feb 12;16(8):9900–7. doi: 10.1021/acsami.3c18855 (PMC10910433; doi:10.1021/acsami.3c18855)
Supplement: Supplementary file 1 — am3c18855_si_001.pdf [file am3c18855_si_001.pdf]

SUPPORTING INFORMATION

Organophosphate detoxification and acetylcholinesterase reactivation triggered by zeolitic imidazolate framework structural degradation

Javier D. Martin-Romera[#,1], Emilio Borrego-Marin[#,1], Pedro J. Jabalera-Ortiz[#], Francesco Carraro,[±] Paolo Falcaro,[±] Elisa Barea[#], Francisco J. Carmona[#,]* and Jorge A. R. Navarro[#,]*

[#]Departamento de Química Inorgánica, Universidad de Granada, Av. Fuentenueva S/N, 18071, Granada, Spain

[±]Institute of Physical and Theoretical Chemistry, TU Gratz, Stremayrgasse 9, A-8010 Graz, Austria

[1]Equal contribution

Corresponding Author

*Francisco J. Carmona: fjcarmona@ugr.es

*Jorge A. R. Navarro: jarn@ugr.es

Index

S.1. Synthesis of compounds. 4

S.1.1. ZIF-20 [Zn(pIm)$_2$] 4

S.1.2. ZIF-11 [Zn(bIm)$_2$] 4

S.1.3. Zn(Im)$_2$ 4

S.1.4. Sod ZIF-8_2 µM [Zn(mIm)$_2$] 4

S.1.5. Sod ZIF-8_1.2 µM [Zn(mIm)$_2$] 5

S.1.6. Sod ZIF-8_240 nm [Zn(mIm)$_2$] 5

S.1.7. Sod ZIF-8_20 nm [Zn(mIm)$_2$] 5

S.1.8. ZIF-L [Zn(mIm)$_2$] 5

S.1.9. ZIF-EC-1 [Zn$_3$(mIm)$_5$(OH)] 5

S.1.10. Zn(2-pymo)$_2$ 6

S.2. Physical and chemical characterization. 6

S.2.1. Elemental analysis. 6

S.2.2. Powder X-Ray Diffraction (PXRD). 7

S.2.3. Fourier Transform Infrared Spectroscopy (FTIR). 10

S.2.4. Thermogravimetric Analysis (TGA) 11

S.2.5. Gas adsorption isotherms. 12

S.2.6. Scanning Electron Microscopy (SEM). 13

S.3. DIFP and DICP degradation studies. 14

S.3.1. Gas Chromatography studies 14

S.3.2. ^{1}H and ^{31}P Nuclear Magnetic Resonance Spectroscopy studies. 17

S.3.3. Scanning Electron Microscopy (SEM) and Transmission Electron Microscopy (TEM) studies. 31

S.3.4. Inductively coupled plasm mass spectroscopy (ICP-MS) 34

S.4. Enzymatic assays. 35

S.4.1. AChE Reactivation assays. 35

S.4.2. Detoxification studies.. 36

S.5. References.. 37

S.1. Synthesis of compounds.

All ZIFs were synthesized as previously reported in literature.

S.1.1. ZIF-20 [Zn(pIm)$_2$]

0.029 g of zinc nitrate hexahydrate (0.10 mmol) were dissolved in 0.40 mL of DMF and 0.06 g of purine (pIm, 0.50 mmol) were dissolved in 0.80 mL of DMF. The two solutions were mixed. Afterwards, 0.80 mL of a triethylamine (TEA) solution in DMF (1.25 mmol of TEA) was added and the mixture was heated in a microwave reactor at 150 ºC during 15 minutes. The resulting precipitate was washed with DMF (3 x 2 mL) and EtOH (3 x 2 mL) and recovered by centrifugation (4000 rpm x 10 min).[1]

S.1.2. ZIF-11 [Zn(bIm)$_2$]

0.12 g of benzimidazole (bIm, 1 mmol) were dissolved in 8.62 mL of EtOH, followed by the addition of 5.31 mL of toluene containing 1 mmol of ammonia. After dissolution, 0.11 g of zinc acetate dyhidrate (0.5 mmol) were added and the solution was stirred during 3 h. The product was washed with EtOH (3 x 10 mL) and recovered by centrifugation (4000 rpm x 10 min).[2]

S.1.3. Zn(Im)$_2$

0.30 g of zinc perchlorate hexahydrate (0.81 mmol) were dissolved in 5 mL of water and 0.11 g of imidazole (Im, 1.61 mmol) were dissolved in 5 mL of water. The two solution were mixed under stirring. Aqueous ammonia was added dropwise until a white precipitate was obtained (pH = 10). The solid was washed with H_2O (3 x 10 mL) and EtOH (1 x 10 mL) and recovered by filtration.[3]

S.1.4. Sod ZIF-8_2 µM [Zn(mIm)$_2$]

0.32 g of zinc acetate dihydrate (1.5 mmol) were dissolved in 10 mL of water and 1.23 g of 2-methylimidazole (mIm, 15 mmol) were dissolved in 20 mL of water. The two solutions were mixed and kept at room temperature overnight. The resulting solid was washed with H_2O (2 x 10 mL) and EtOH (1 x 10 mL) and recovered by centrifugation (3500 rpm x 5 min).[4]

S.1.5. Sod ZIF-8_1.2 µM [Zn(mIm)$_2$]

0.32 g of zinc acetate dihydrate (1.5 mmol) were dissolved in 10 mL of water and 2.46 g of 2-methylimidazole (mIm, 30 mmol) were dissolved in 20 mL of water. The two solutions were mixed and kept at room temperature overnight. The resulting solid was washed with H_2O (2 x 10 mL) and EtOH (1 x 10 mL) and recovered by centrifugation (3500 rpm x 5 min).[4]

S.1.6. Sod ZIF-8_240 nm [Zn(mIm)$_2$]

0.32 g of zinc acetate dihydrate (1.5 mmol) were dissolved in 10 mL of water and 4.92 g of 2-methylimidazole (mIm, 60 mmol) were dissolved in 20 mL of water. The two solutions were mixed and kept at room temperature overnight. The resulting solid was washed with H_2O (2 x, 10 mL) and EtOH (1 x 10 mL) and recovered by centrifugation (3500 rpm x 5 min).[4]

S.1.7. Sod ZIF-8_20 nm [Zn(mIm)$_2$]

0.73 g of zinc nitrate hexahydrate (2.5 mmol) were dissolved in 50 mL of methanol and 0.81 g of 2-methylimidazole (mIm, 9.9 mmol) together with 0.975 mL of n-butylamine (9.9 mmol) were dissolved in 50 mL of methanol. The latter solution was poured into the former under stirring and the mixture was kept at room temperature during 24 h. The resulting solid was washed with MeOH (3 x 25 mL) and recovered by centrifugation (15,000 rpm x 5 min).[4]

S.1.8. ZIF-L [Zn(mIm)$_2$]

0.59 g of zinc nitrate hexahydrate (2 mmol) were dissolved in 40 mL of water and 1.24 g of 2-methylimidazole (mIm, 15 mmol) were dissolved in 40 mL of water. The two solutions were mixed and stirred during 24 hours at room temperature. The resulting solid was washed with water (3 x 20 mL) and recovered by centrifugation (4000 rpm, 10 min).[5]

S.1.9. ZIF-EC-1 [Zn$_3$(mIm)$_5$(OH)]

0.05 g of zinc acetate dihydrate (0.29 mmol) were dissolved in 1 mL of water and 0.04 g of 2-methylimidazole (mIm, 0.48 mmol) were dissolved in 0.125 mL of water. The two solutions were mixed and stirred during 4 hours at room temperature. The resulting white

powder was washed with water (6 x 1 mL) and recovered by centrifugation (12 000 rpm, 2 min).[6]

S.1.10. Zn(2-pymo)$_2$

1.36 g of $ZnCl_2$ (10 mmol) and 3.31 g of 1H-pyrimidin-2-one (34 mmol) were dissolved in 30 mL of water. After 20 minutes of stirring, we heated at 90 ºC and added ammonia solution up to pH = 10 and kept stirring 30 additional minutes at 90 ºC. The resulting white solid was filtered, washed with warm H_2O (3 x 10 mL) and dry under vacuum.[7]

S.2. Physical and chemical characterization.

S.2.1. Elemental analysis.

Table S1. Elemental analysis of sod ZIF-20, ZIF-11, Zn(Im)$_2$, ZIF-L, ZIF-EC-1 and sod ZIFs-8 (2 µm, 1 µm, 240 nm and 20 nm)**.**

	Theoretical			Experimental		
	N (%)	C (%)	H (%)	N (%)	C (%)	H (%)
ZIF-20	30.20	32.38	2.17	30.19	33.49	3.89
ZIF-11	18.57	55.74	4.01	16.14	59.75	5.18
Zn(Im)$_2$	27.80	35.76	4.00	27.78	35.50	3.77
ZIF-L	24.62	42.21	4.43	24.75	39.87	6.72
ZIF-EC-1	22.46	38.51	5.00	20.76	37.25	5.84
Sod ZIF-8_2 µm	24.62	42.21	4.43	24.51	42.14	6.34
Sod ZIF-8_1.2 µm	24.62	42.21	4.43	25.74	43.74	6.36
Sod ZIF-8_240 nm	24.62	42.21	4.43	25.19	42.89	6.02
Sod ZIF-8_20 nm	24.62	42.21	4.43	23.88	41.13	5.76

S.2.2. Powder X-Ray Diffraction (PXRD).

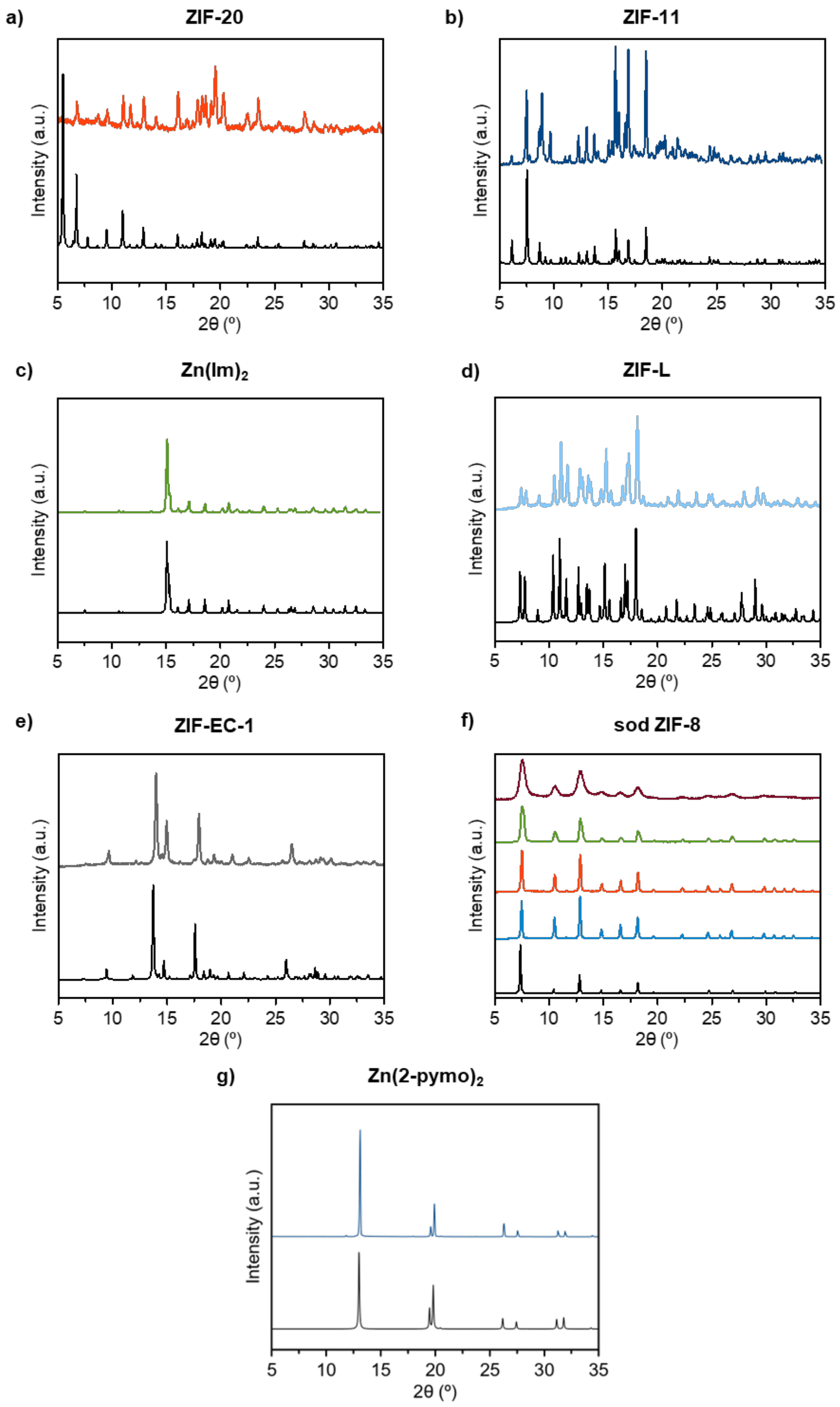

Figure S1. PXRD of (a) **ZIF-20**, (b) **ZIF-11**, (c) **Zn(Im)$_2$**,(d) **ZIF-L**, (e) **ZIF-EC-1** and (f) **sod ZIFs-8** (2 µm, blue line; 1 µm, red line; 240 nm, green line and 20 nm, brown line) (g) **Zn(2-pymo)$_2$**. The simulated patterns are drawn in black.

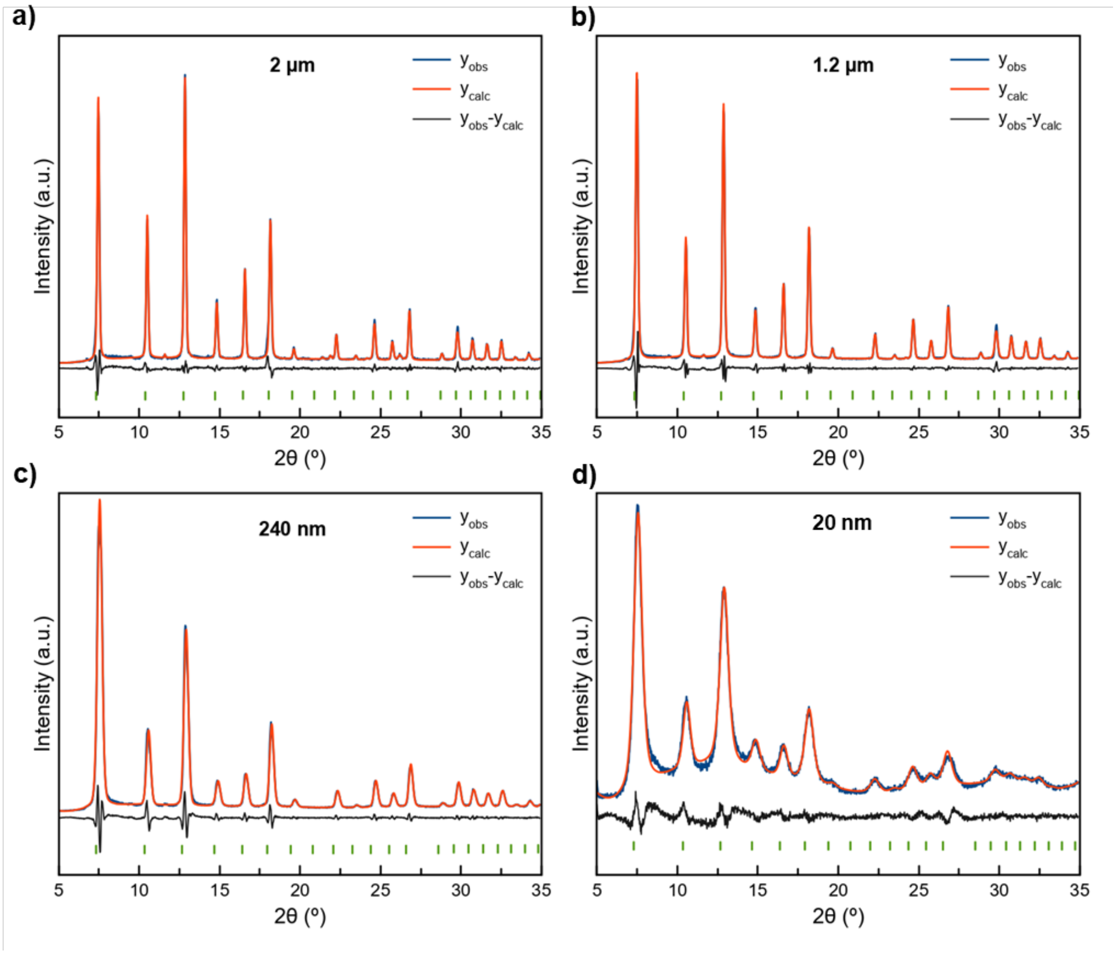

Figure S2. Graphical result of the whole powder pattern refinement carried out with the Le Bail method on the PXRD pattern of as-synthesized sod ZIF-8 particles of decreasing size in terms of observed, calculated and difference traces (blue, red and grey, respectively). The positions of the Bragg reflection are indicated by green ticks. Sod ZIFs-8 particles of 2 µm (a); 1 µm (b); 240 nm (c); and 20 nm (d).

Table S2. Cell parameters for sod ZIFs-8 particles determined by PXRD Le Bail analysis. Crystalline domain size was estimated using a Lorentzian-convolution of Bragg peaks performed with the software Topas v3.

ZIF-8	Cell Parameter (Å)	Error (Å)	CS_L (nm)	Error_L (nm)
20 nm	17.125	0.006	24	1
240 nm	17.086	0.009	186	24
1.2 um	17.0224	0.0007	202	7
2 um	17.0339	0.0009	215	8

S.2.3. Fourier Transform Infrared Spectroscopy (FTIR).

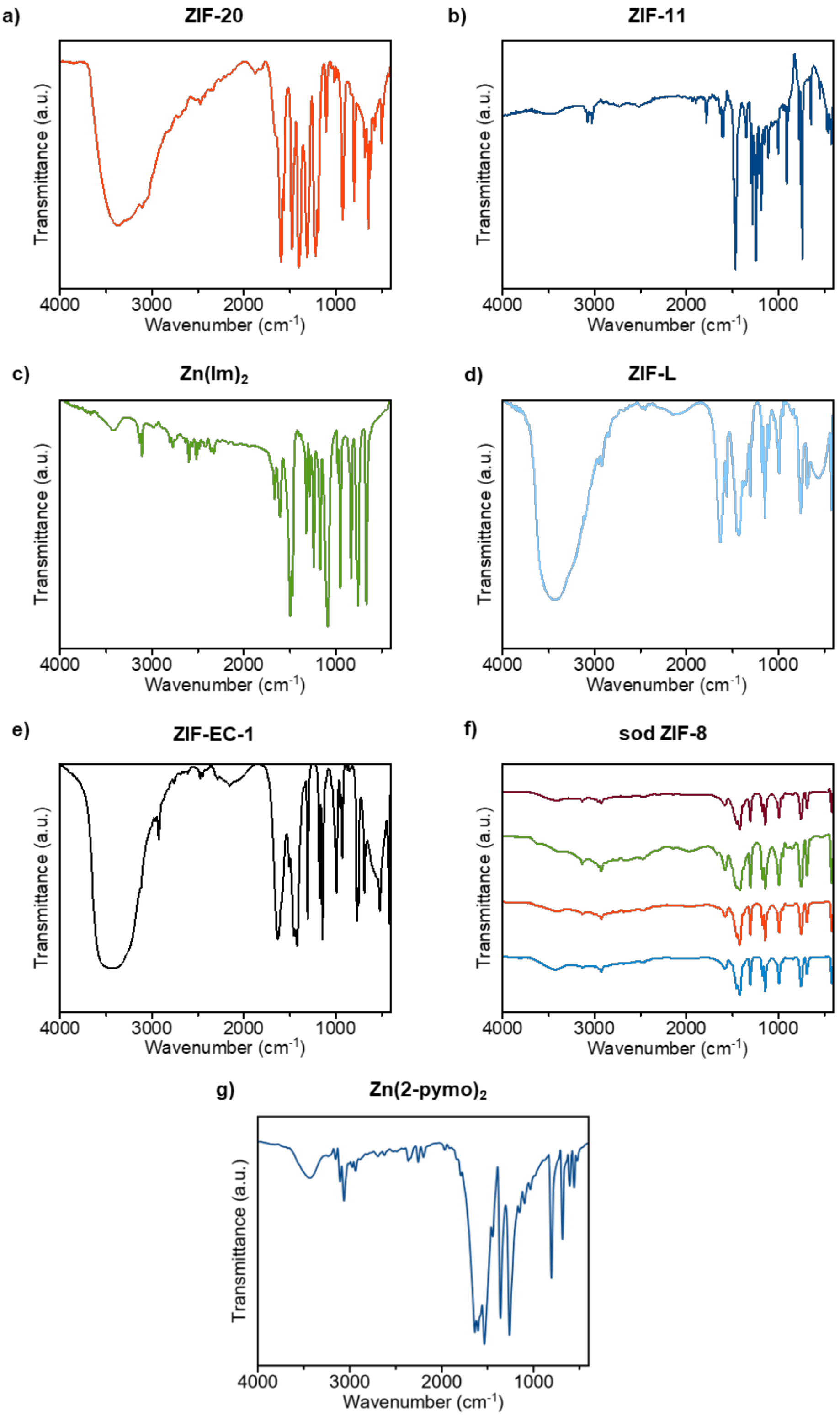

Figure S3. Infra-red spectra of (a) **ZIF-20**, (b) **ZIF-11**, (c) **$Zn(Im)_2$**,(d) **ZIF-L**, (e) **ZIF-EC-1** and (f) **sod ZIFs-8** (2 µm, blue line; 1 µm, red line; 240 nm, green line and 20 nm, brown line) (g) **$Zn(2\text{-}pymo)_2$**.

S.2.4. Thermogravimetric Analysis (TGA)

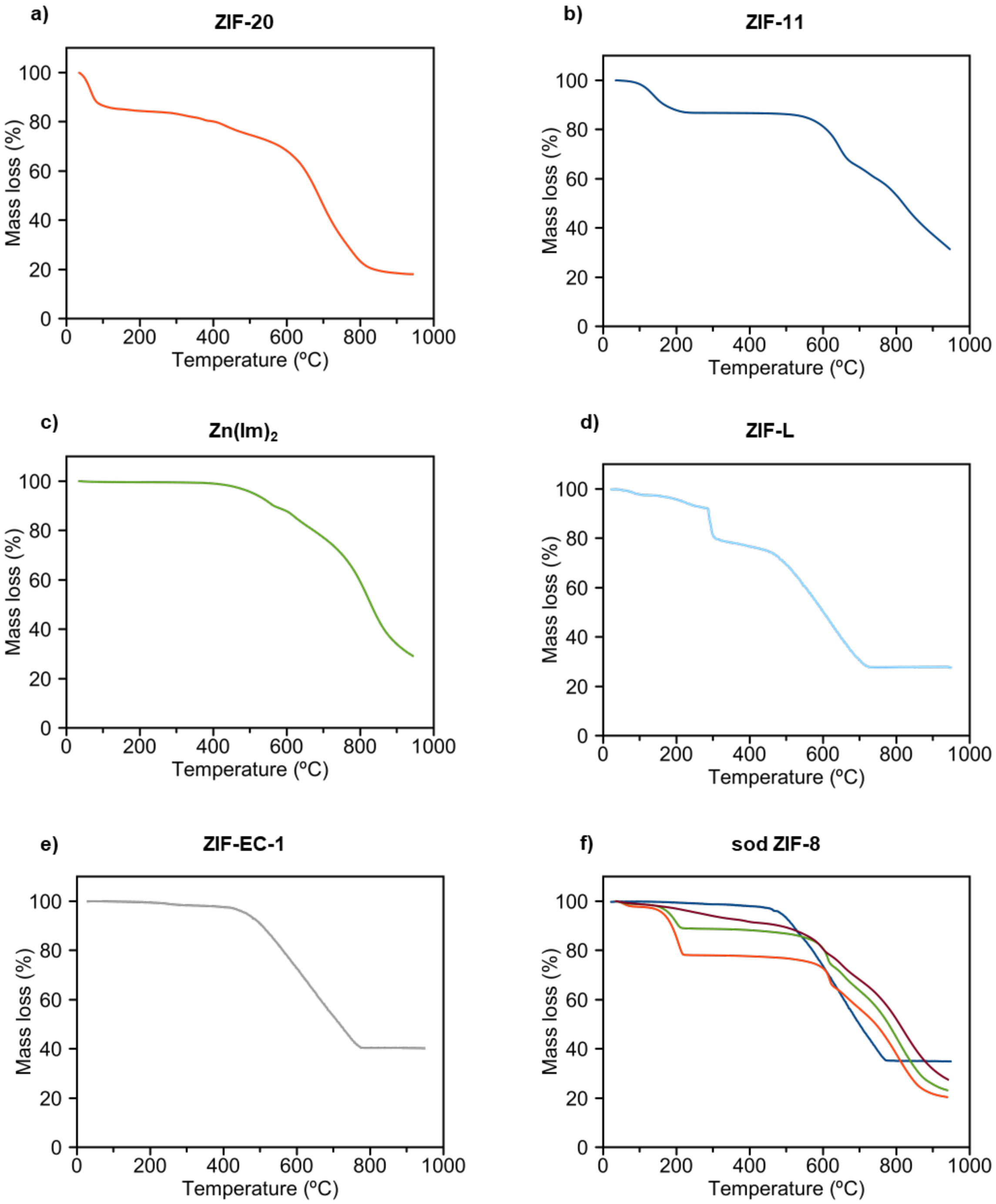

Figure S4. Thermogravimetric analysis of studied ZIFs under air atmosphere (20 mL min^{-1}) and heating ramp of 10 ºC min^{-1} for a) ZIF-20, b) ZIF-11, c) Zn(Im)2, d) ZIF-L, e) ZIF-EC-1, f) sod ZIF-8 particles 2µm (red trace), 1.2µm (green trace), 240 nm (brown trace), 20 nm (blue trace).

S.2.5. Gas adsorption isotherms.

Prior to nitrogen adsorption measurements, different sod ZIFs-8 were soaked in MeOH during 24 h. This process was performed by triplicate. Afterwards, the materials were activated at 100 ºC during 12 h under dynamic vacuum.

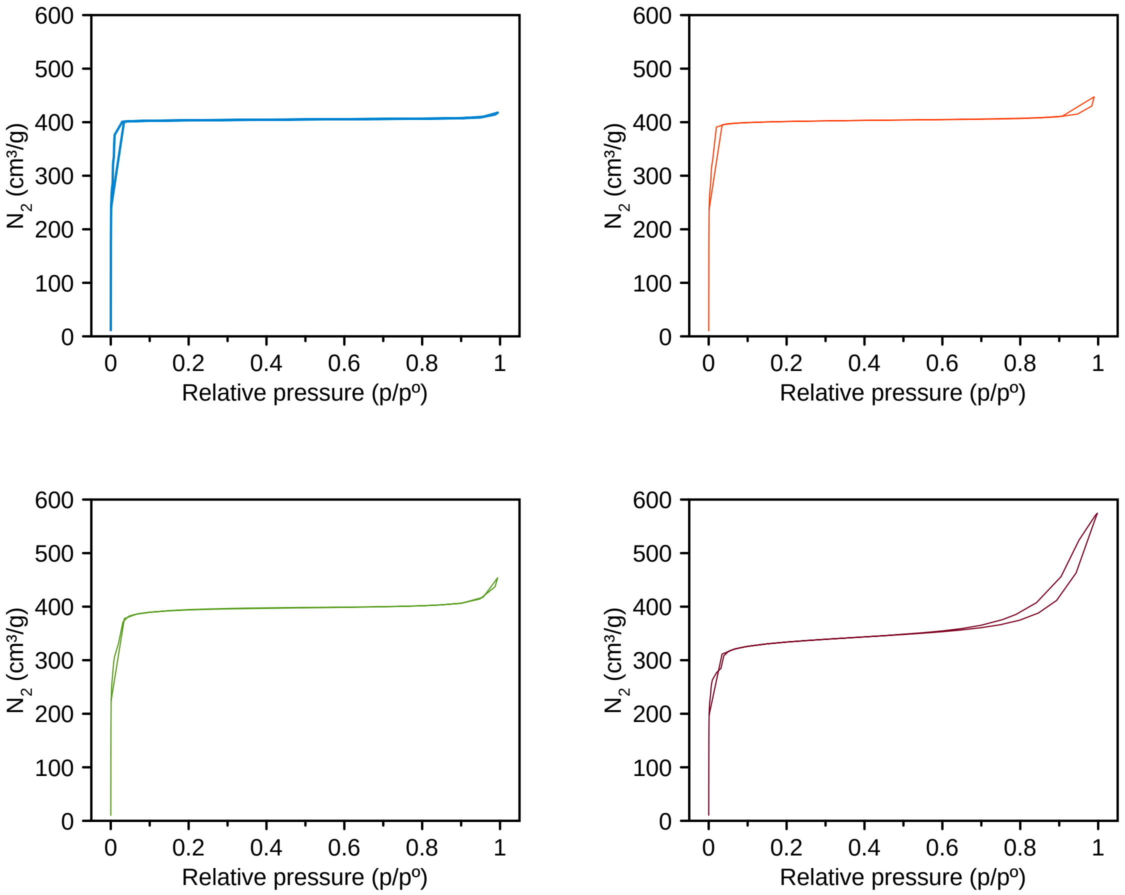

Figure S5. Nitrogen adsorption isotherm of (a) **sod ZIF-8_2 µm** (BET = 1624 $m^2\ g^{-1}$), (b) **sod ZIF-8_1.2 µm** (BET = 1622 $m^2\ g^{-1}$), (c) **sod ZIF-8_240 nm** (BET= 1620 $m^2\ g^{-1}$) and (d) **sod ZIF-8_20 nm** (BET = 1540 $m^2\ g^{-1}$).

S.2.6. Scanning Electron Microscopy (SEM).

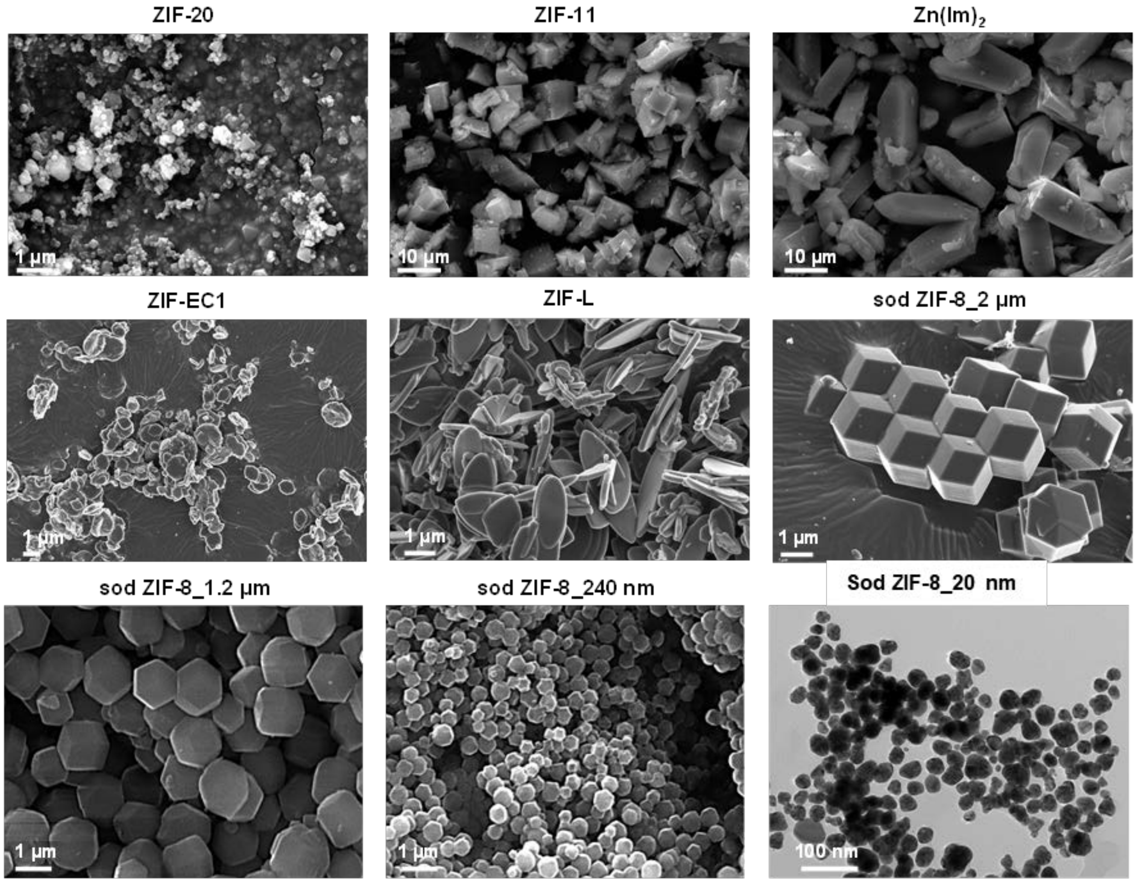

Figure S6. SEM and TEM images of **ZIF-20**, **ZIF-11**, **Zn(Im)$_2$**, **ZIF-EC1, ZIF-L**, **sod ZIF-8_2 nm**, **sod ZIF-8_1.2 µm**, **sod ZIF-8_240 nm** and **sod ZIF-8_20 nm**

S.3. DIFP and DICP degradation studies.

S.3.1. Gas Chromatography studies

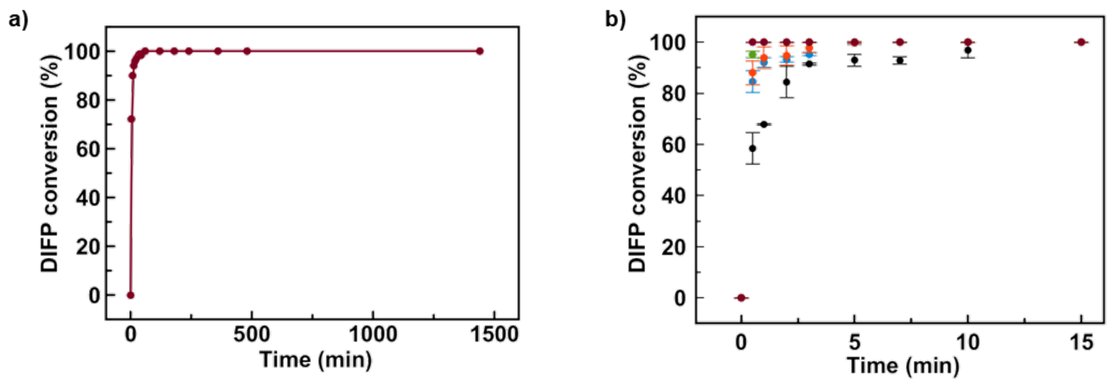

Figure S7. a) Profile of DIFP (0.029 M, 0.5 mL) hydrolytic degradation by sod **ZIF-8** 20 nm (0.084 mmol), in phosphate buffered saline solution (0.01 M, pH 7.4) to evaluate the possible phosphate interference; b) Profile of DICP (0.029 M, 0.5 mL) hydrolytic degradation by 0.084 mmol sod **ZIF-8** of different particle size 2 µm (cyan), 1.2 µm (red), 240 nm (green), 20 nm (brown), control (black) under simulated biological conditions (Tris-DCl, 0.1 M, pD 7.8).

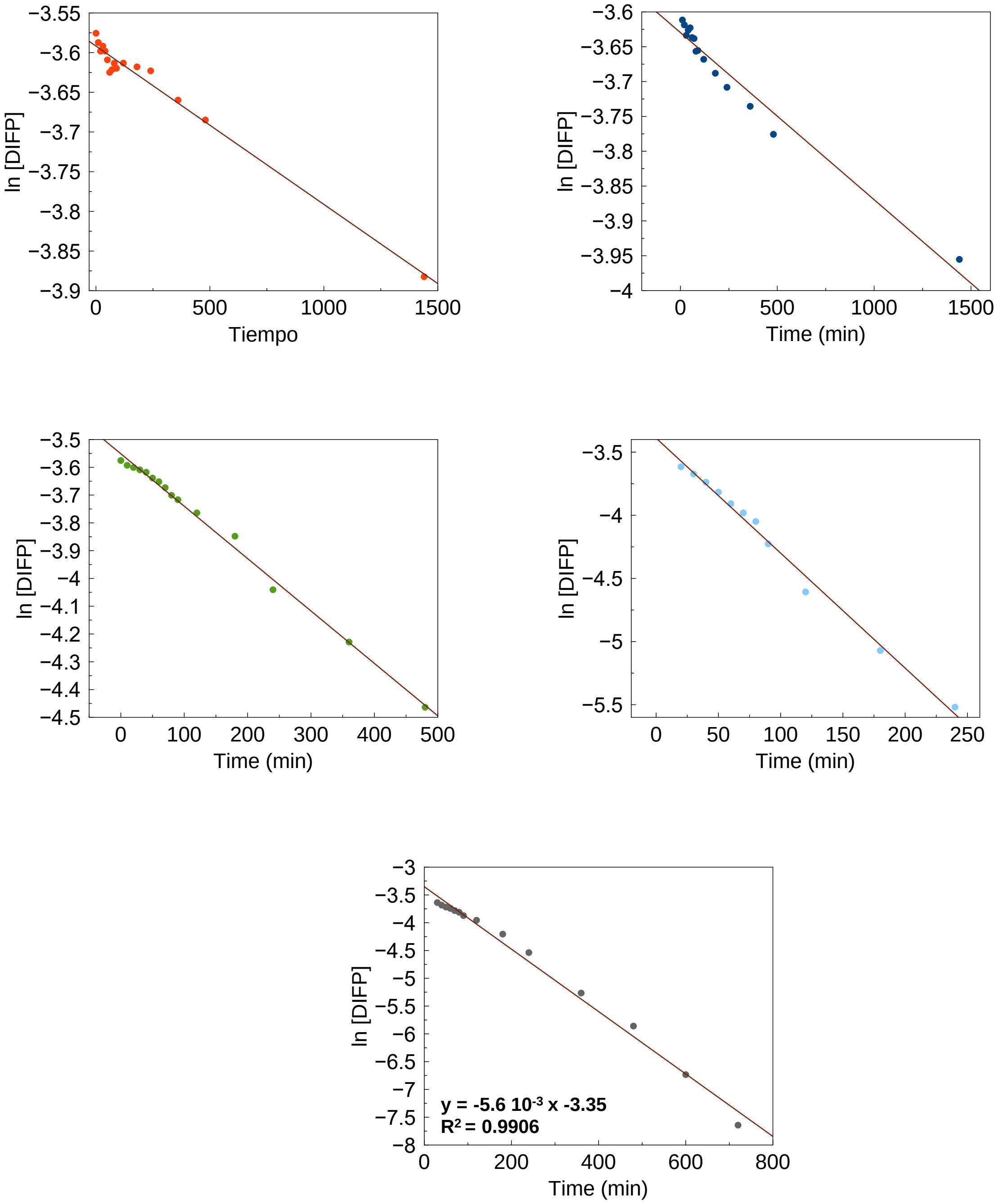

Figure S8. Fitting curves to first-order kinetic model of DIFP hydrolytic degradation (0.029 M, 0.5 mL) under simulated biological conditions by (a) **ZIF-20** (0.084 mmol, (b) **ZIF-11** (0.084 mmol), (c) **Zn(Im)$_2$** (0.084 mmol), (d) **ZIF-L** (0.084 mmol) and (e) **ZIF-EC-1** (0.029 mmol).

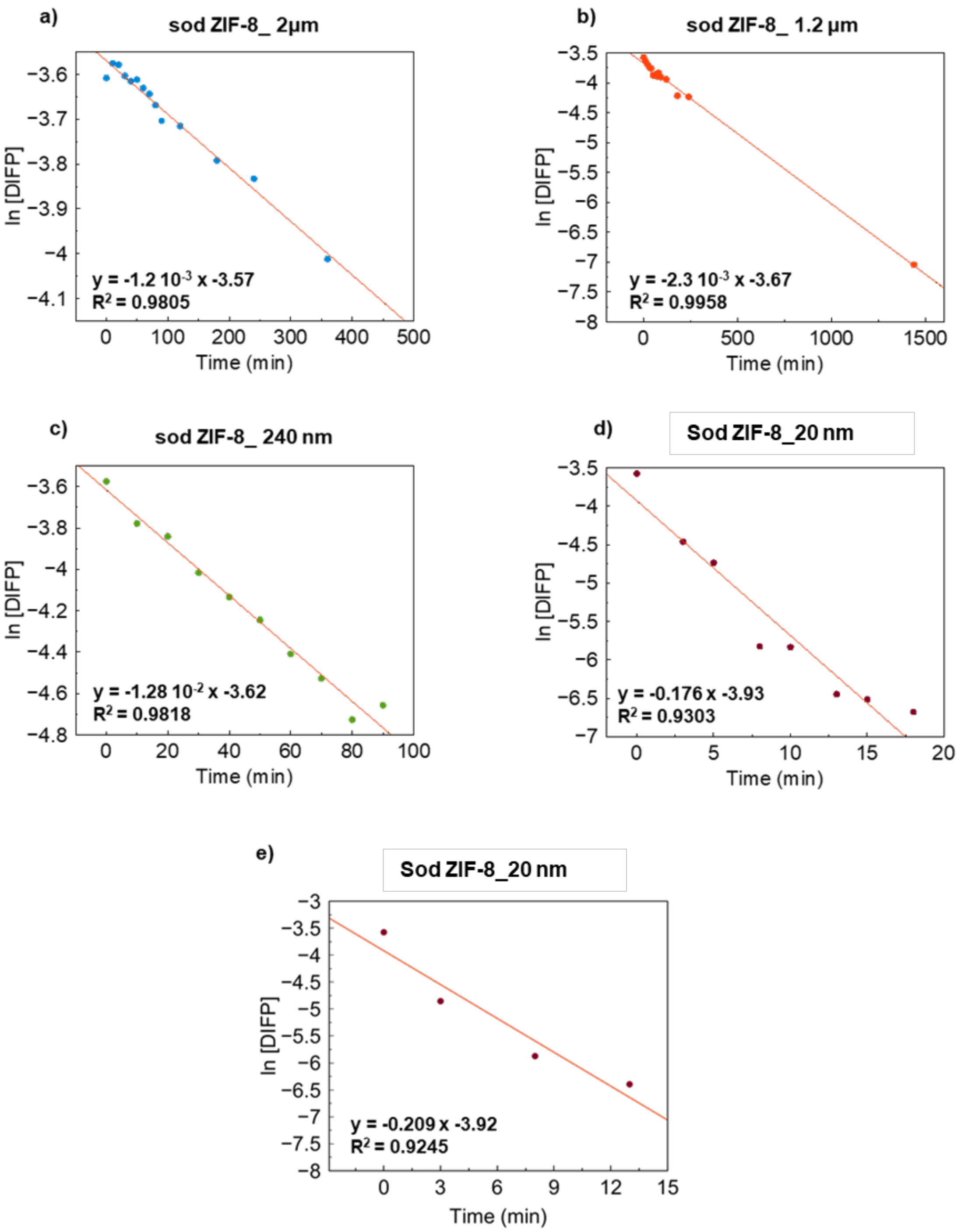

Figure S9. Fitting curves to first-order kinetic model of DIFP hydrolytic degradation (0.029 M, 0.5 mL) under simulated biological conditions by (a) **sod ZIF-8_2 μm** (0.084 mmol, (b) **sod ZIF-8_1.2 μm** (0.084 mmol), (c) **sod ZIF-8_240 nm** (0.084 mmol) and (d) **sod ZIF-8_20 nm** (0.084 mmol) in Tris-HCl (0.1 M, pH 7.4) and (e) **sod ZIF-8_20 nm** (0.084 mmol) in phosphate buffered saline solution (0.01 M, pH 7.4).

S.3.2. ¹H and ³¹P Nuclear Magnetic Resonance Spectroscopy studies.

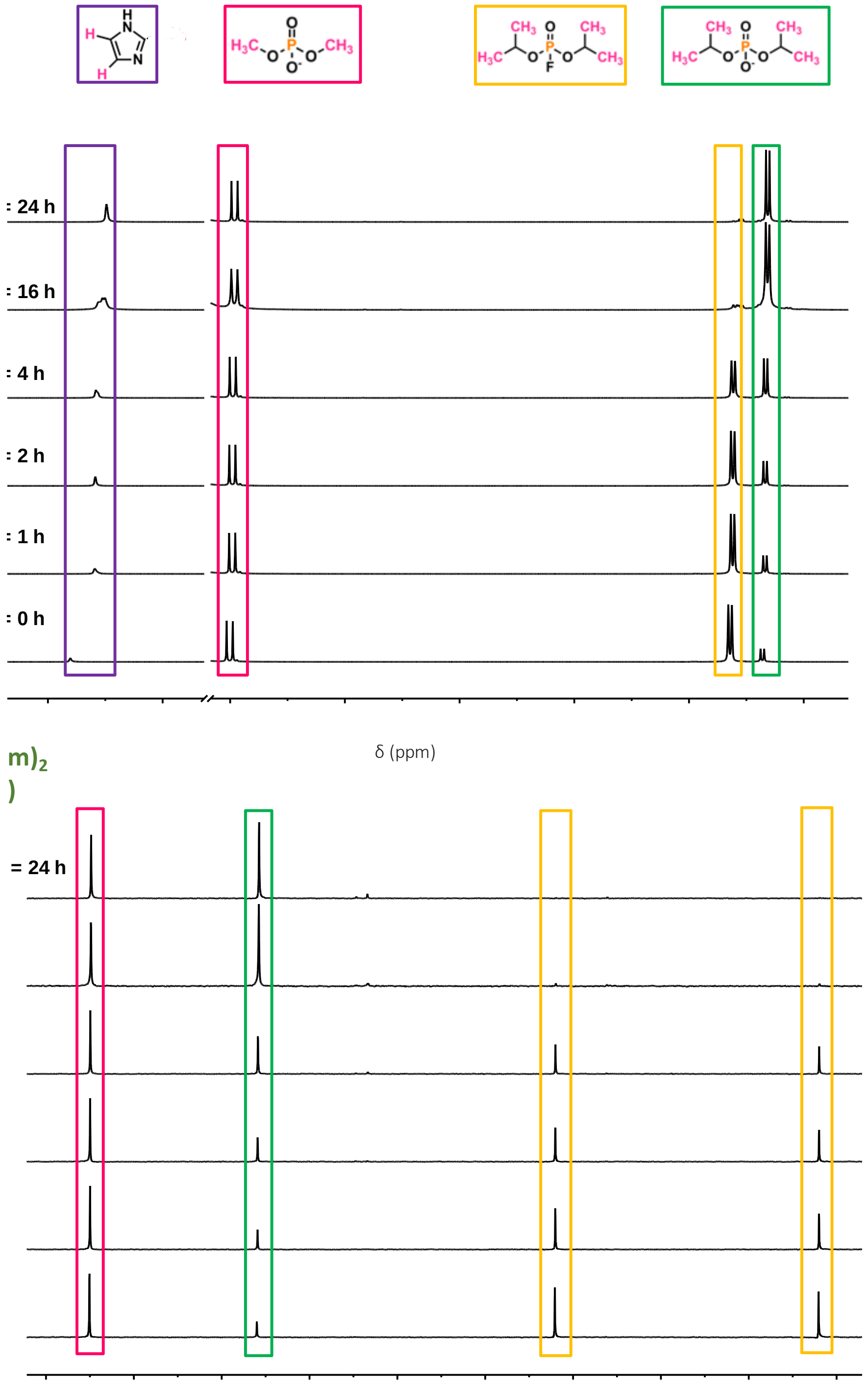

Figure S10. ¹H NMR spectra (top) and ³¹P NMR spectra (bottom) of hydrolytic DIFP degradation by **Zn(Im)₂**. Experimental conditions: DIFP (0.029 M), **Zn(Im)₂** (0.084 mmol), dimethylphosphate (0.029 M, internal reference), Tris-DCl (0.1 M, pD 7.8, 0.5 mL), room temperature.

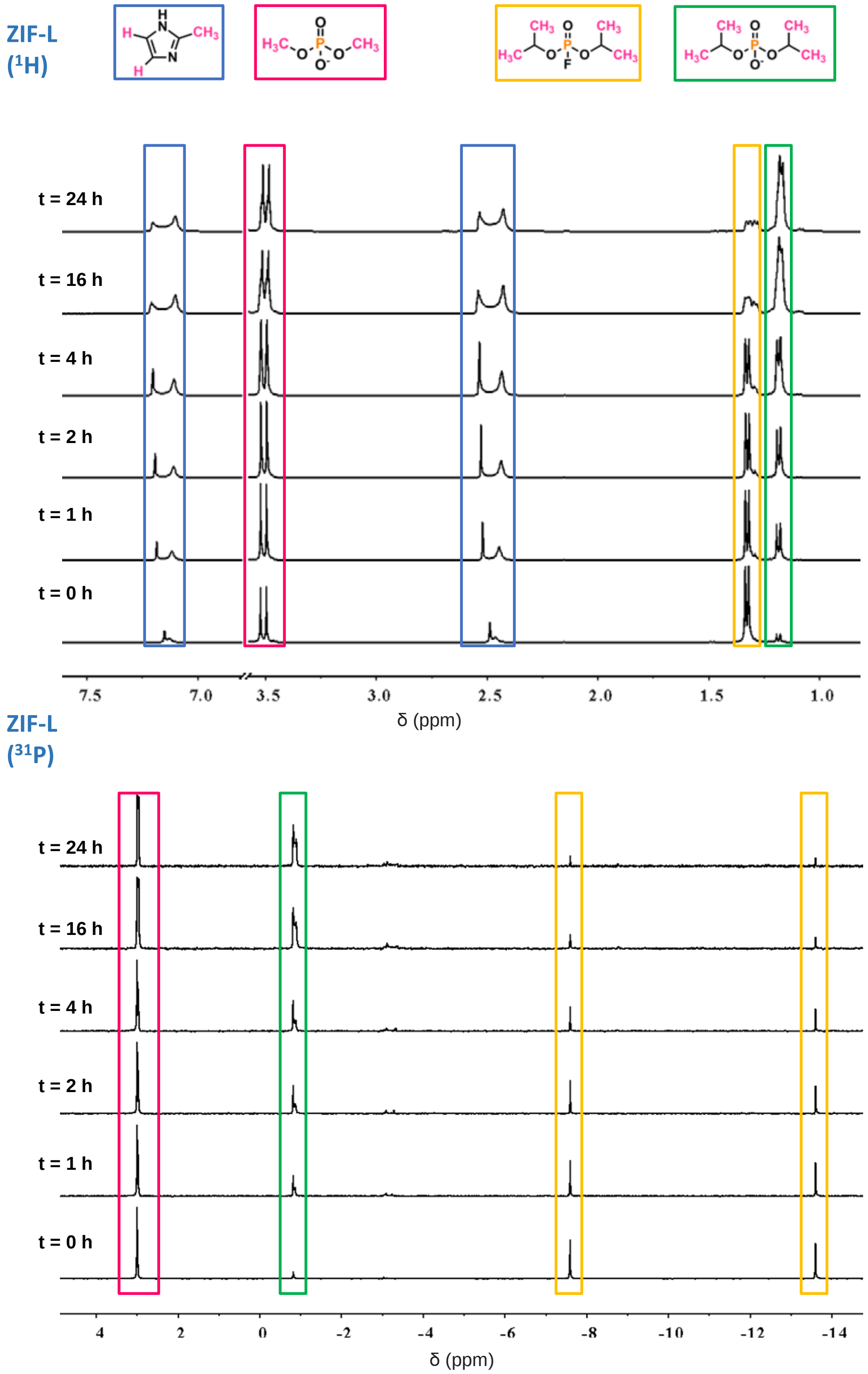

Figure S11. ^{1}H NMR spectra (top) and ^{31}P NMR spectra (bottom) of hydrolytic DIFP degradation by **ZIF-L**. Experimental conditions: DIFP (0.029 M), **ZIF-L** (0.084 mmol), dimethylphosphate (0.029 M, internal reference), Tris-DCl (0.1 M, pD 7.8, 0.5 mL), room temperature.

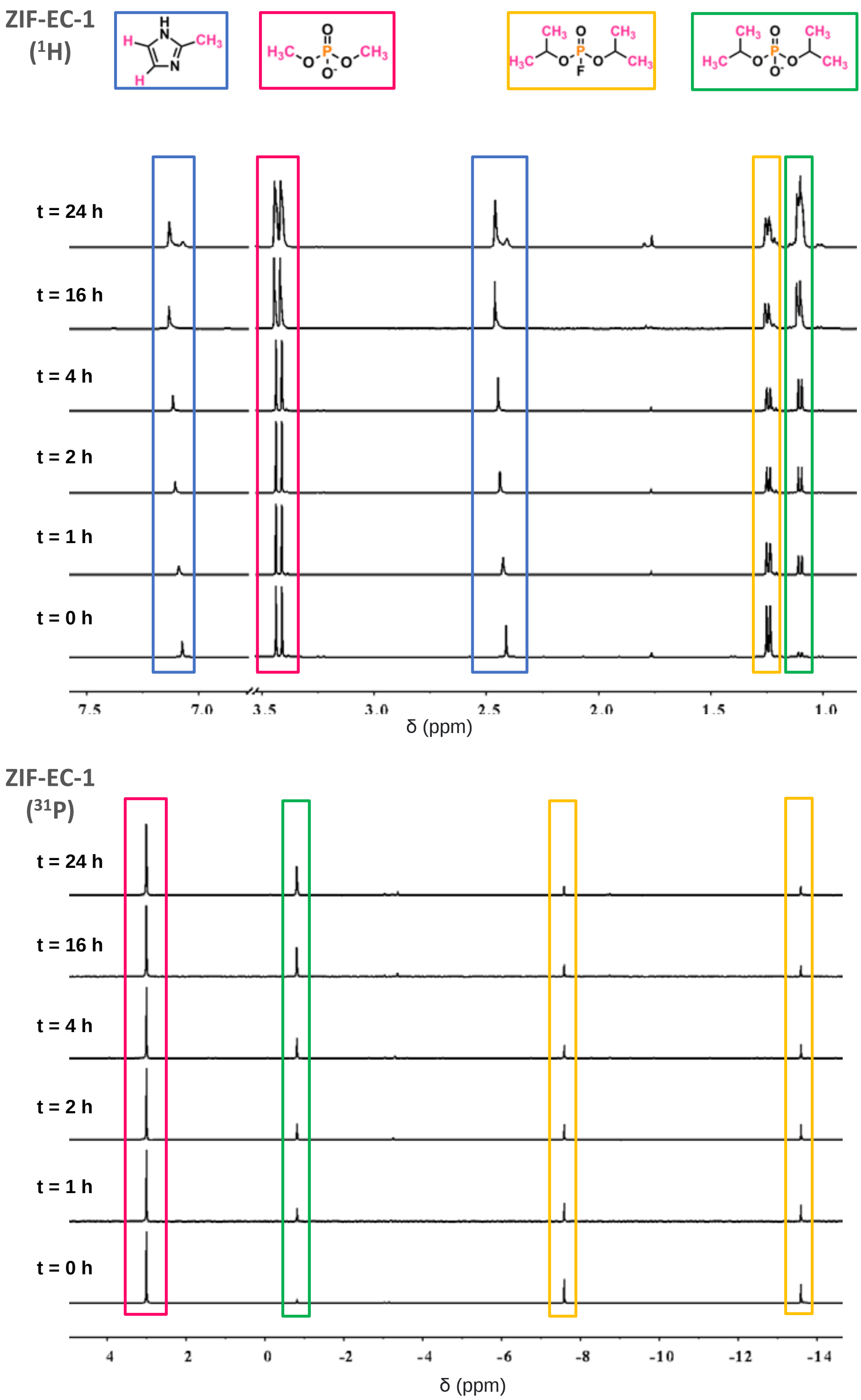

Figure S12. ^{1}H NMR spectra (top) and ^{31}P NMR spectra (bottom) of the DIFP degradation studies by **ZIF-EC-1**. Experimental conditions: DIFP (0.029 M), **ZIF-EC-1** (0.029 mmol), dimethylphosphate (0.029 M, internal reference), and Tris-DCl (0.1 M, pD 7.8, 0.5 mL), room temperature.

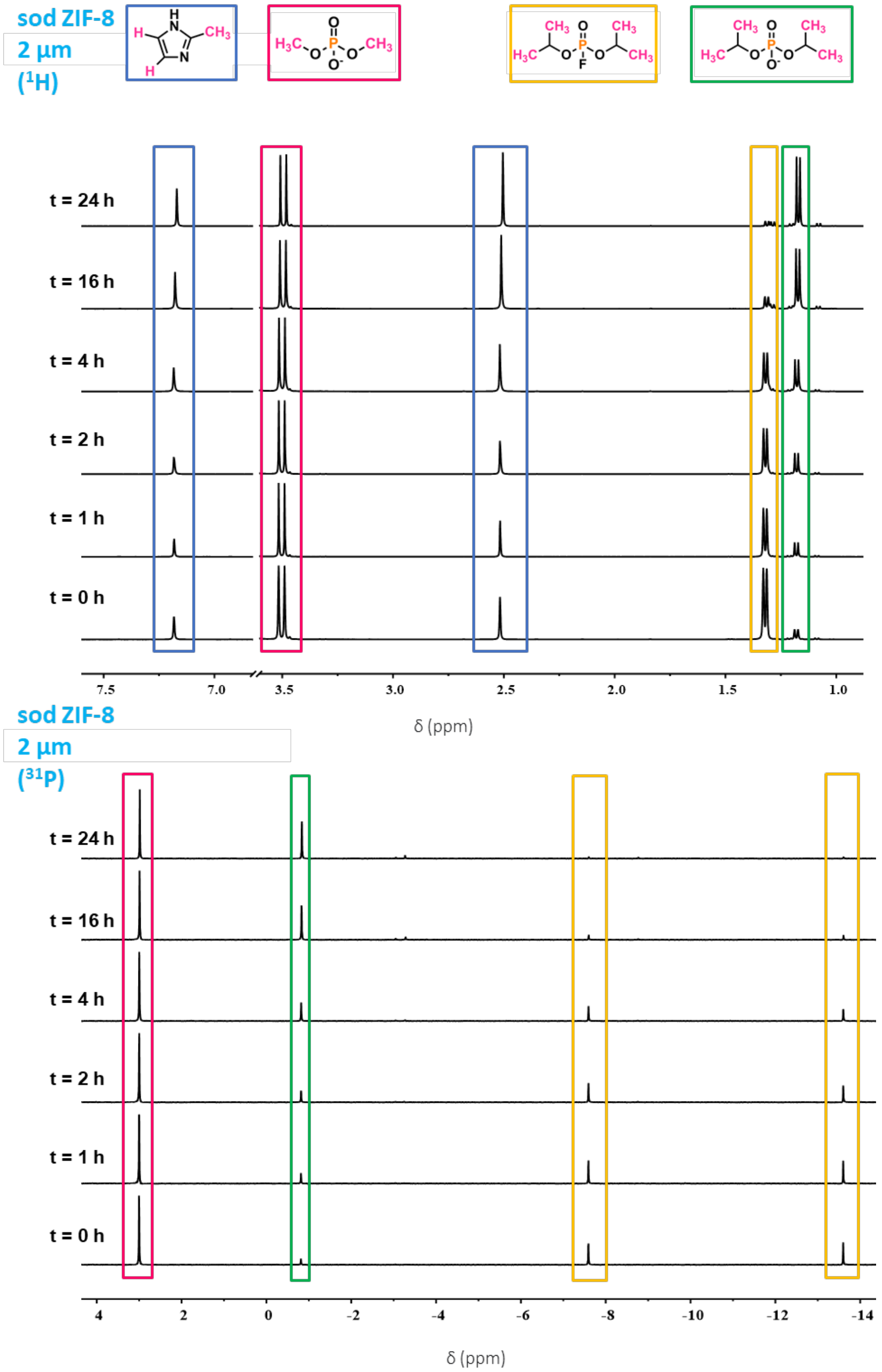

Figure S13. ^{1}H NMR spectra (top) and ^{31}P NMR spectra (bottom) of the DIFP degradation studies by **sod ZIF-8_2 µm**. Experimental conditions: DIFP (0.029 M), **sod ZIF-8_2 µm** (0.084 mmol), dimethylphosphate (0.029 M, internal reference), Tris-DCl (0.1 M, pD 7.8, 0.5 mL), room temperature.

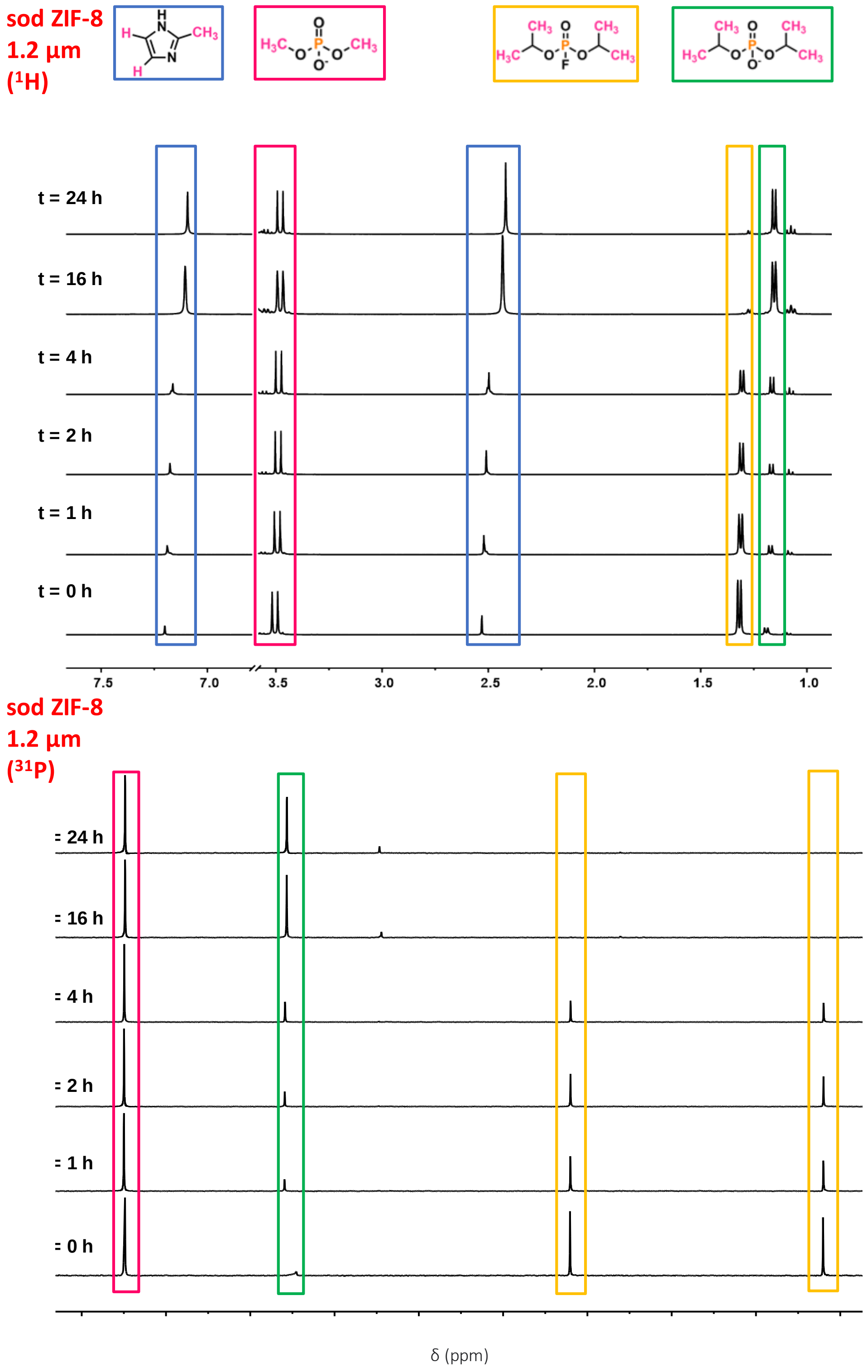

Figure S14. ^{1}H NMR spectra (top) and ^{31}P NMR spectra (bottom) of the DIFP degradation studies by **sod ZIF-8_1.2 µm**. Experimental conditions: DIFP (0.029 M), **sod ZIF-8_1.2 µm** (0.084 mmol), dimethylphosphate (0.029 M, internal reference), Tris-DCl (0.1 M, pD 7.8, 0.5 mL), room temperature.

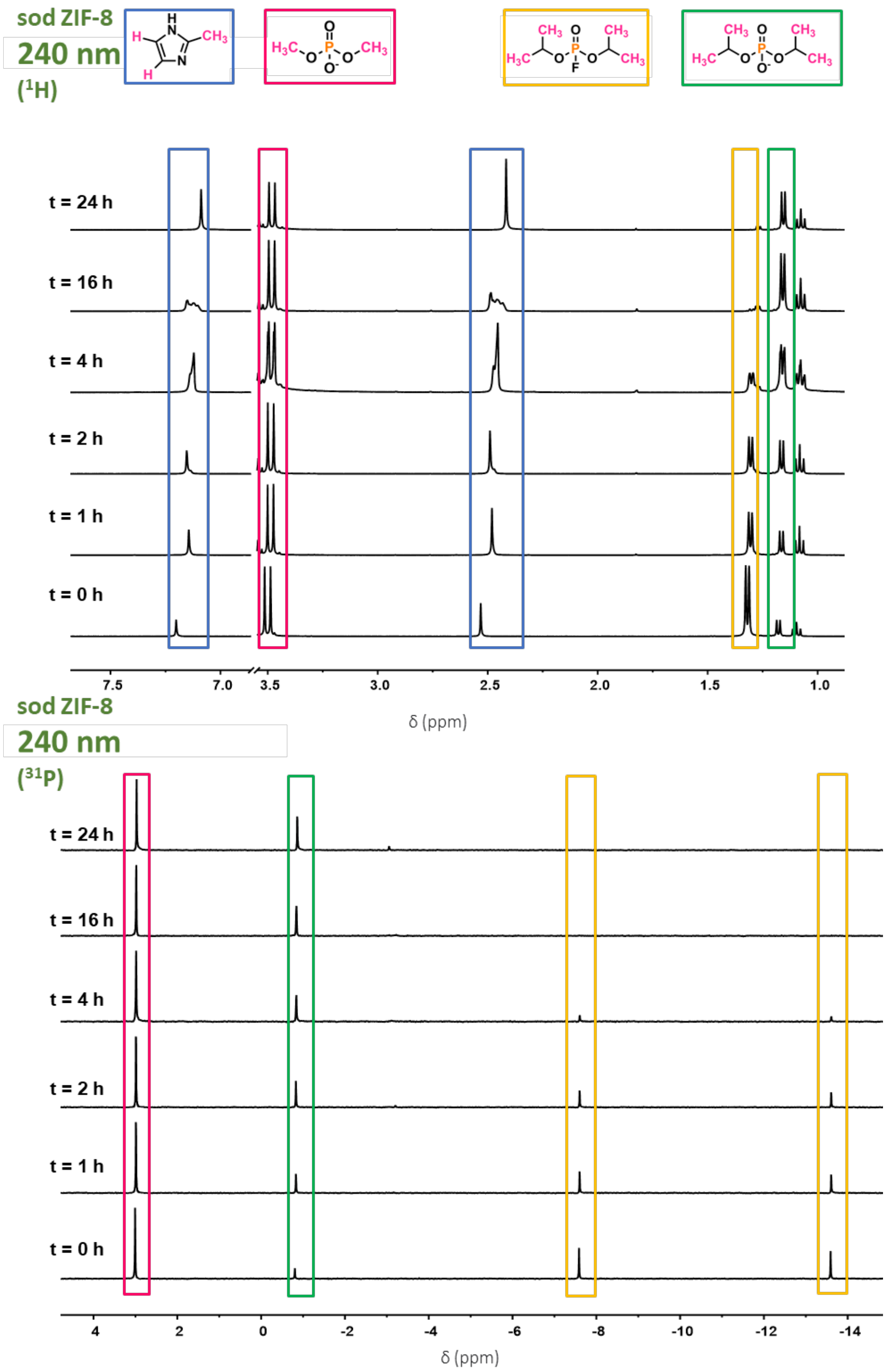

Figure S15. ^{1}H NMR spectra (top) and ^{31}P NMR spectra (bottom) of the DIFP degradation studies by **sod ZIF-8_240 nm**. Experimental conditions: DIFP (0.029 M), **sod ZIF-8_240 nm** (0.084 mmol), dimethylphosphate (0.029 M, internal reference), Tris-DCl (0.1 M, pD 7.8, 0.5 mL), room temperature.

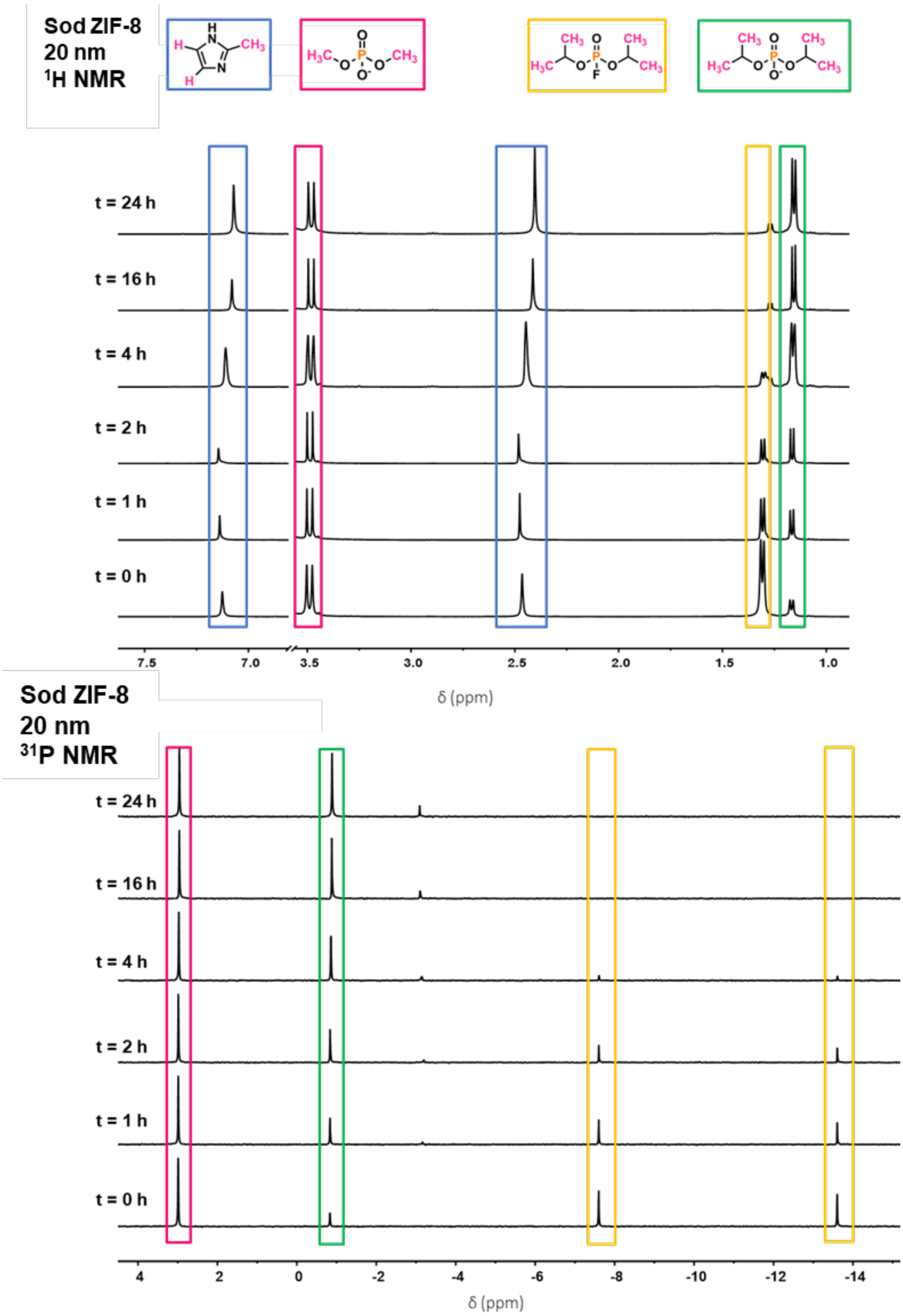

Figure S16. ^{1}H NMR spectra (top) and ^{31}P NMR spectra (bottom) of the DIFP degradation studies by **sod ZIF-8_20 nm**. Experimental conditions: DIFP (0.029 M), **sod ZIF-8_20 nm** (0.084 mmol), dimethylphosphate (0.029 M, internal reference), Tris-DCl (0.1 M, pD 7.8, 0.5 mL), room temperature.

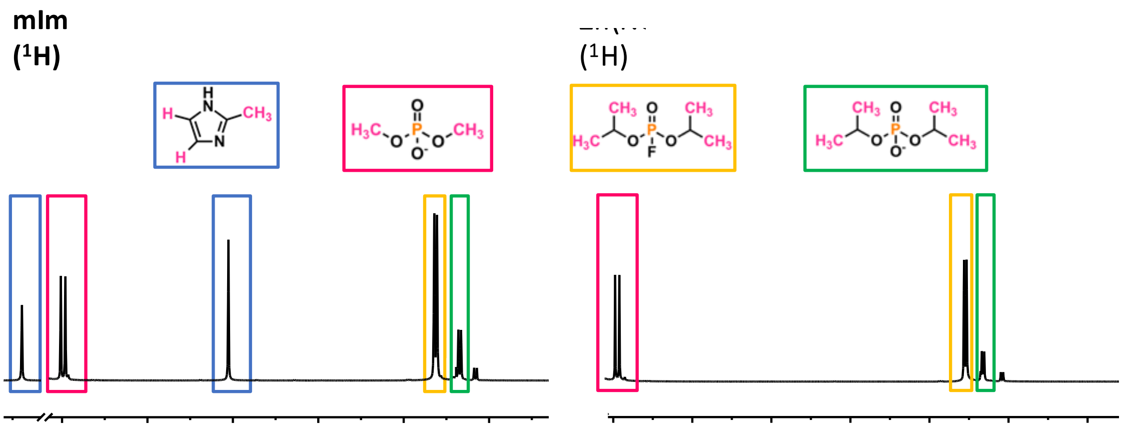

Figure S17. ^{1}H NMR spectra of the DIFP degradation studies by **2-methylimidazole (mIm)** (left) and **Zn(NO$_3$)$_2$** (right) after 24 h. Experimental conditions: DIFP (0.029 M), **Zn(NO$_3$)$_2$** (0.10 mmol), **2-methylimidazole** (0.02 mmol), dimethylphosphate (0.029 M, internal reference), Tris-DCl (0.1 M, pD 7.8, 0.5 mL), room temperature.

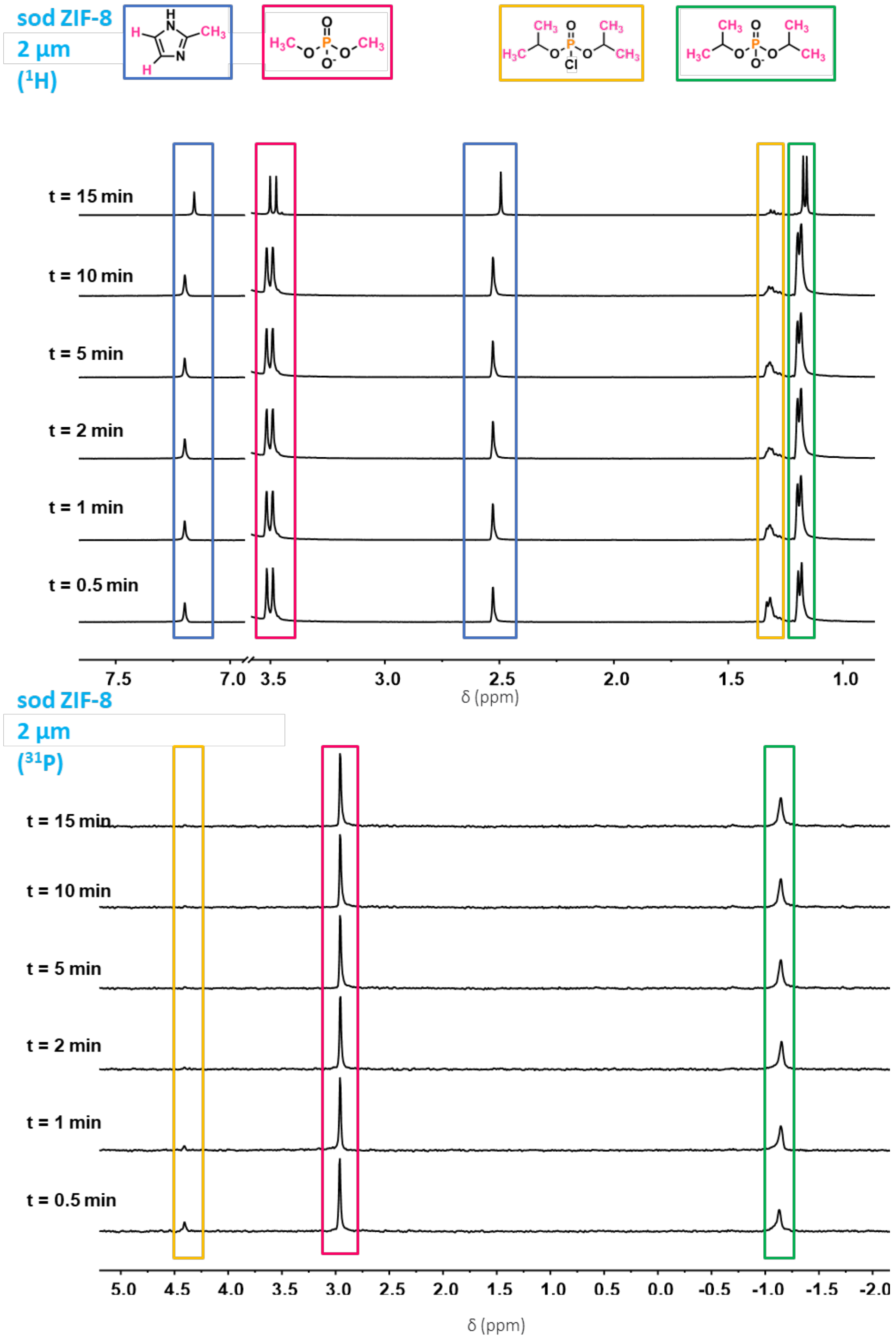

Figure S18. ^{1}H NMR spectra (top) and ^{31}P NMR spectra (bottom) of the DICP degradation studies by **sod ZIF-8_2 μm**. Experimental conditions: DICP (0.029 M), **sod ZIF-8_2 μm** (0.084 mmol), dimethylphosphate (0.029 M, internal reference), Tris-DCl (0.1 M, pD 7.8, 0.5 mL), room temperature.

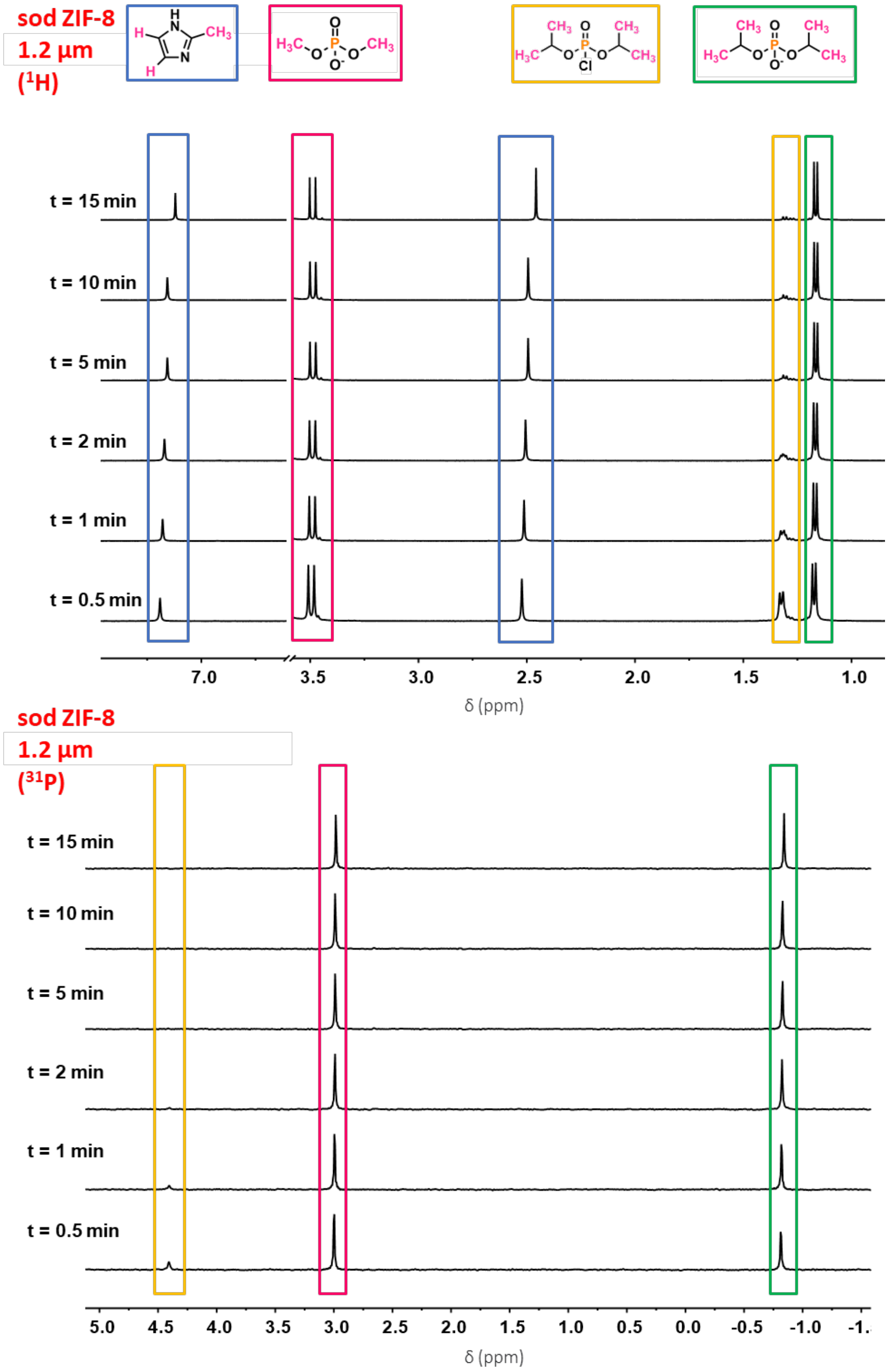

Figure S19. ^{1}H NMR spectra (top) and ^{31}P NMR spectra (bottom) of the DICP degradation studies by **sod ZIF-8_1.2 µm**. Experimental conditions: DICP (0.029 M), **sod ZIF-8_1.2 µm** (0.084 mmol), dimethylphosphate (0.029 M, internal reference), Tris-DCl (0.1 M, pD 7.8, 0.5 mL), room temperature.

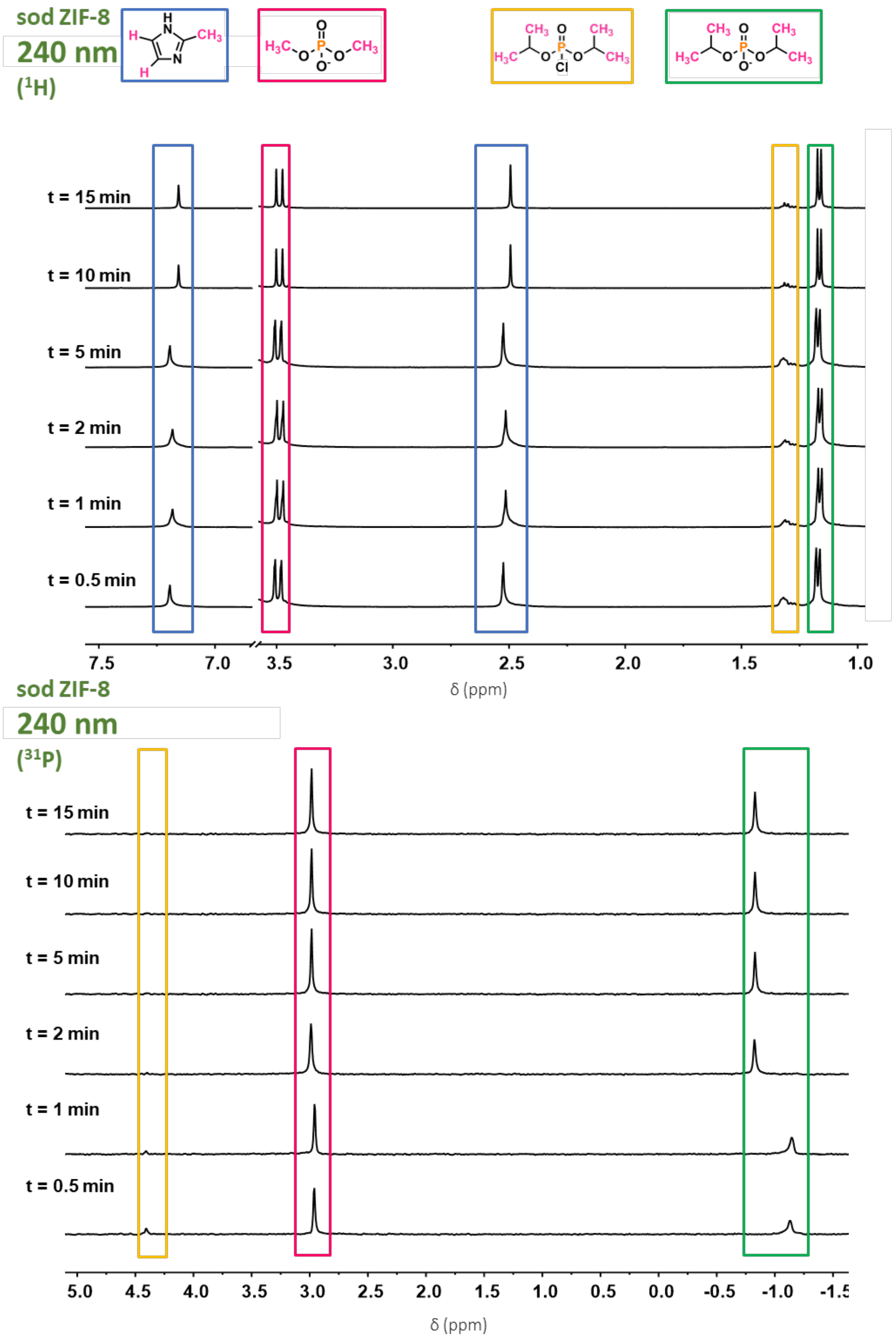

Figure S20. ^{1}H NMR spectra (top) and ^{31}P NMR spectra (bottom) of the DICP degradation studies by **sod ZIF-8_240 nm**. Experimental conditions: DICP (0.029 M), **sod ZIF-8_240 nm** (0.084 mmol), dimethylphosphate (0.029 M, internal reference), Tris-DCl (0.1 M, pD 7.8, 0.5 mL), room temperature.

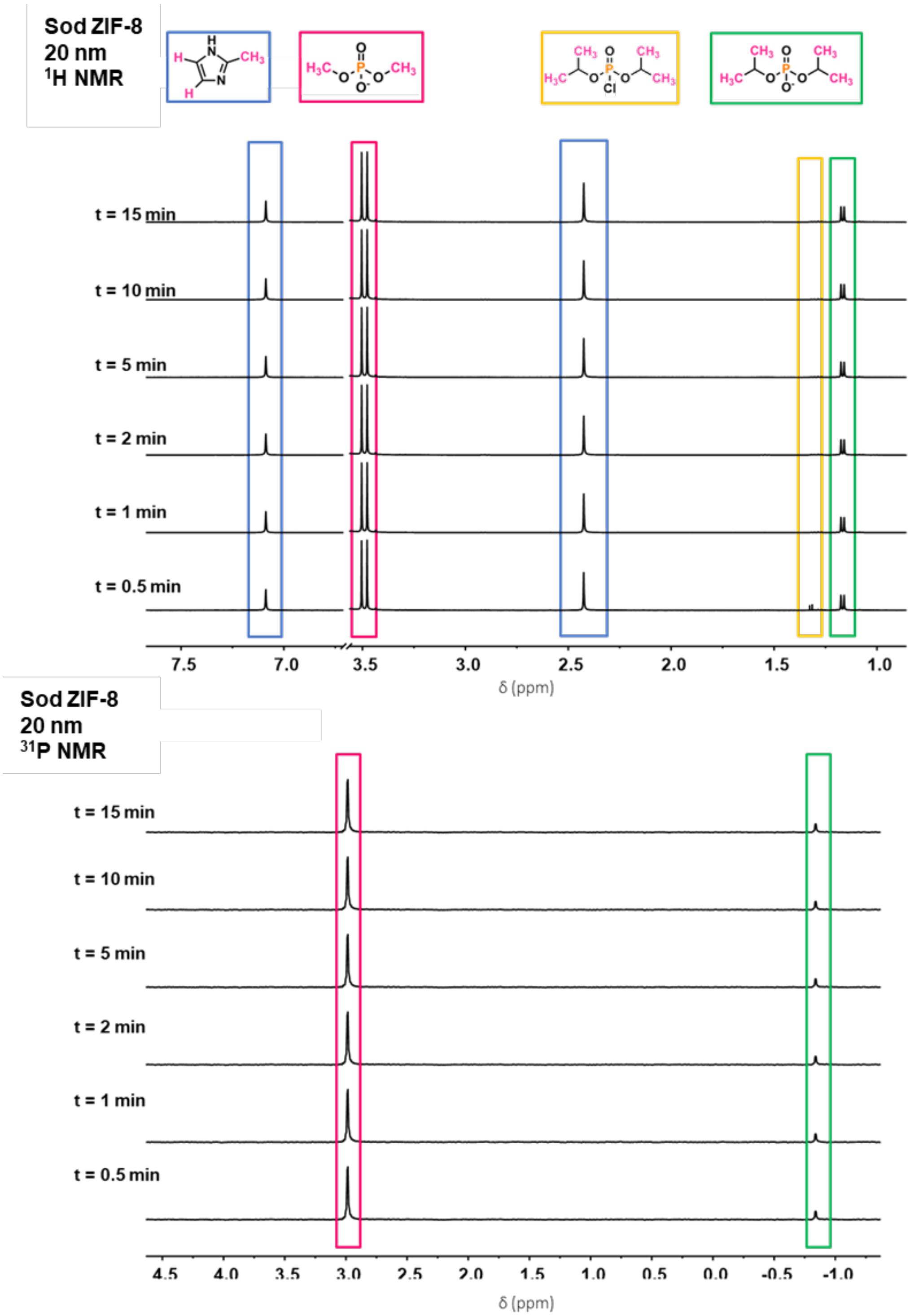

Figure S21. ^{1}H NMR spectra (top) and ^{31}P NMR spectra (bottom) of the DICP degradation studies by **sod ZIF-8_20 nm**. Experimental conditions: DICP (0.029 M), **sod ZIF-8_20 nm** (0.084 mmol), dimethylphosphate (0.029 M, internal reference), Tris-DCl (0.1 M, pD 7.8, 0.5 mL), room temperature.

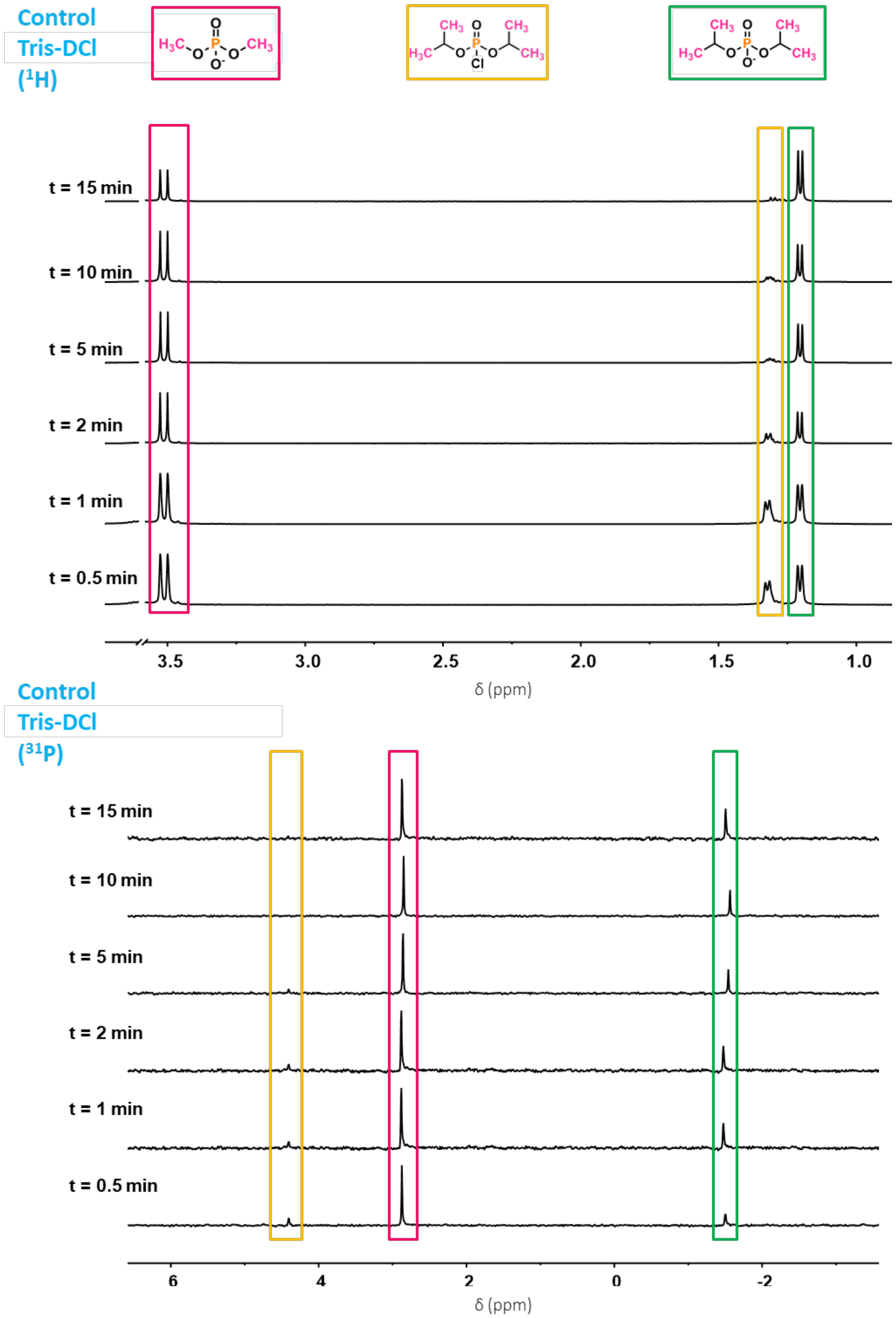

Figure S22. Control experiment of DICP stability under simulated biological conditions analysed by ^{1}H NMR spectra (top) and ^{31}P NMR spectra (bottom). Experimental conditions: DICP (0.029 M), dimethylphosphate (0.029 M, internal reference), Tris-DCl (0.1 M, pD 7.8, 0.5 mL), room temperature.

Table S3. Release of imidazolate linker after ZIF degradation exposed to Tris-HCl (0.1 M, pH 7.4) or to a solution of DIFP (0.029 M) in Tris-HCl (0.1 M, pH 7.4). Experimental conditions: DIFP (0.029 M), ZIF-20 (0.084 mmol), ZIF-11 (0.084 mmol), $Zn(Im)_2$ (0.084 mmol), ZIF-L (0.084 mmol), ZIF-EC-1 (0.029 mmol), sod ZIF-8_2 µm (0.084 mmol), sod ZIF-8_1.2 µm (0.084 mmol), sod ZIF-8_240 nm (0.084 mmol), sod ZIF-8_20 nm (0.084 mmol), Tris-HCl (0.1 M, pH 7.4, 0.5 mL), room temperature.

Sample	DIFP treatment Imidazolate linker release (M)	Tris-HCl treatment Imidazolate linker release (M)
**ZIF-20**	5.0×10^{-3}	3.4×10^{-3}
**ZIF-11**	1.7×10^{-3}	0
**$Zn(Im)_2$**	2.4×10^{-3}	0
**ZIF-L**	5.7×10^{-2}	8.7×10^{-3}
**ZIF-EC-1**	2.2×10^{-2}	6.0×10^{-3}
**Sod ZIF-8_2 µm**	4.2×10^{-2}	2×10^{-2}
**Sod ZIF-8_1.2 µm**	5.1×10^{-2}	3.3×10^{-2}
**Sod ZIF-8_240 nm**	3.5×10^{-2}	2.5×10^{-2}
**Sod ZIF-8_20 nm**	4.5×10^{-2}	1.2×10^{-2}

Table S4. Release of imidazolate linker after sod ZIF-8 degradation exposed to a solution of DICP (0.029 M) in Tris-DCl (0.1 M, pD 7.8, 0.5 mL). Experimental conditions: DICP (0.029 M), sod ZIF-8_2 µm (0.084 mmol), sod ZIF-8_1.2 µm (0.084 mmol), sod ZIF-8_240 nm (0.084 mmol), sod ZIF-8_20 nm (0.084 mmol), room temperature.

Sample	DICP treatment 2-mIm linker release (M)
**Sod ZIF-8_2 µm**	2.52×10^{-2}
**Sod ZIF-8_1.2 µm**	4.95×10^{-2}
**Sod ZIF-8_240 nm**	3.91×10^{-2}
**Sod ZIF-8_20 nm**	2.47×10^{-2}

S.3.3. Scanning Electron Microscopy (SEM) and Transmission Electron Microscopy (TEM) studies.

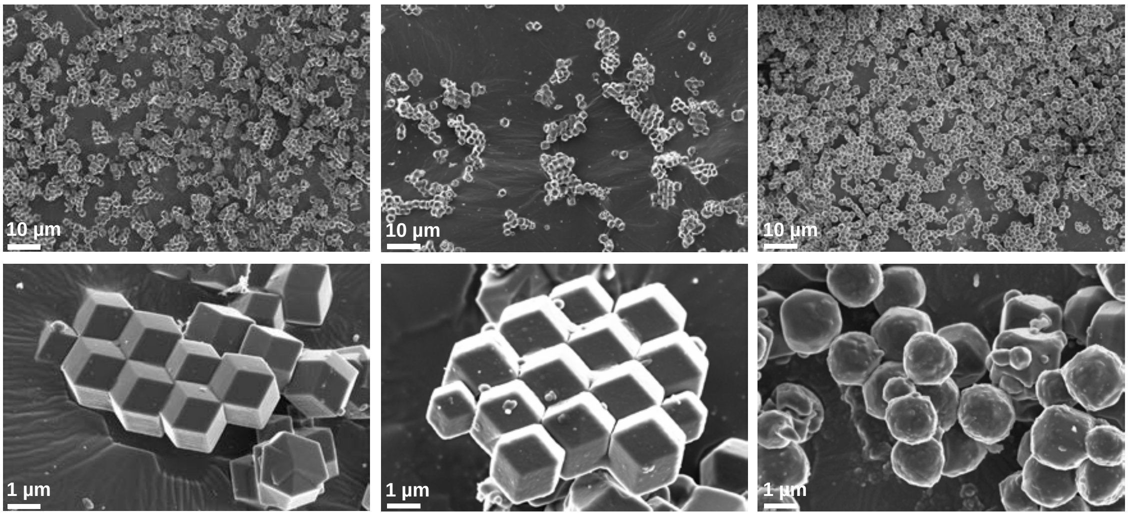

Figure S23. SEM image of (left) pristine **sod ZIF-8** particles, (center) **sod ZIF-8** particles incubated in Tris-HCl for 24 hours and (right) **sod ZIF-8** particles incubated with Tris-HCl and DIFP for 24 hours. Experimental conditions: **sod ZIF-8** (0.015 mmol), Tris-HCl (0.1 M, pH 7.4, 0.5 mL) and DIFP (0.18 M), room temperature.

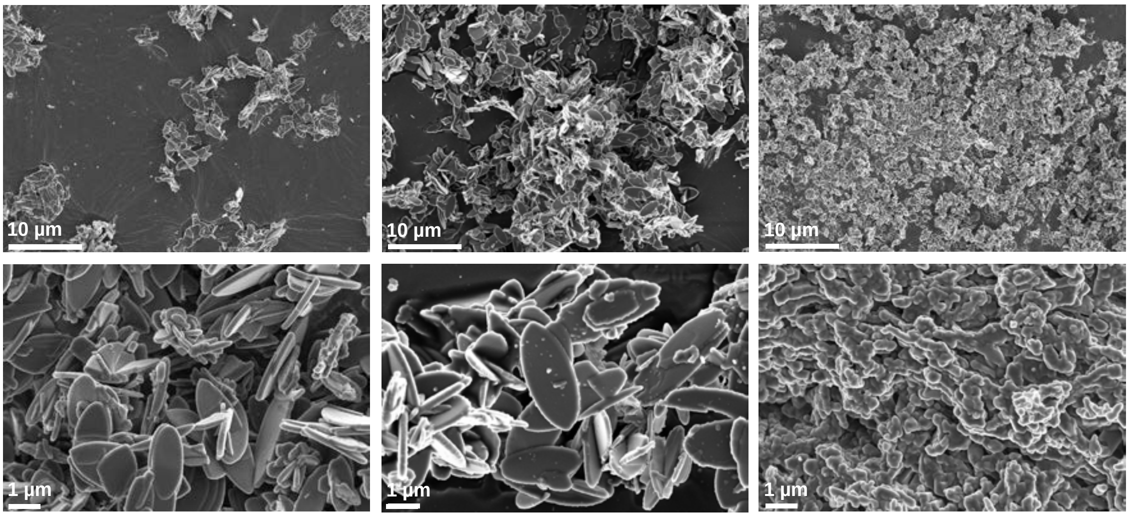

Figure S24. SEM image of (left) pristine **ZIF-L** particles, (center) **ZIF-L** particles incubated with Tris-HCl during 24 hours and (right) **ZIF-L** particles incubated with Tris-HCl and DIFP during 24 hours. Experimental conditions: **ZIF-L** (0.015 mmol), Tris-HCl (0.1 M, pH 7.4, 0.5 mL) and DIFP (0.18 M), room temperature.

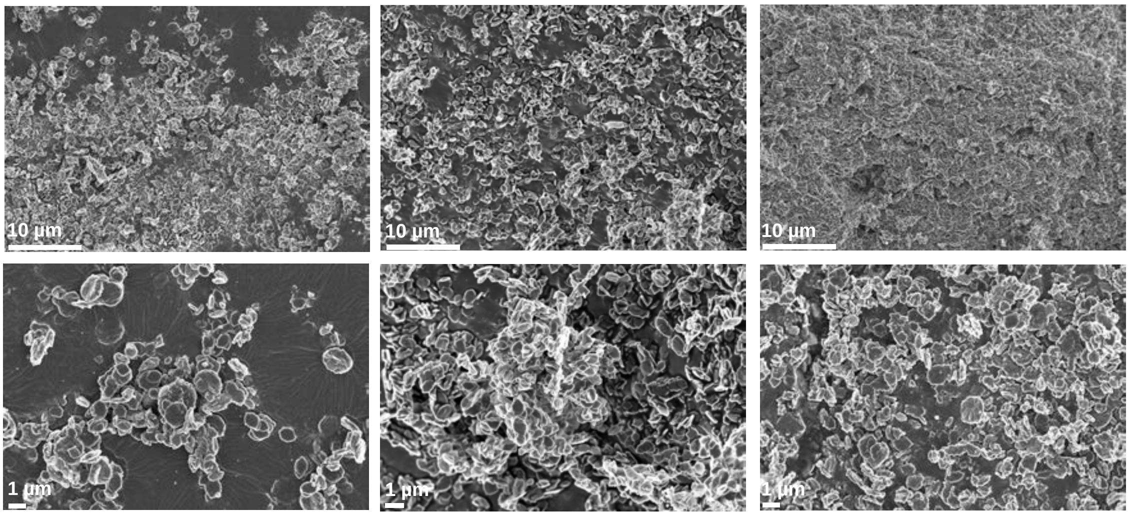

Figure S25. SEM image of (left) pristine **ZIF-EC-1** particles, (center) **ZIF-EC-1** particles incubated with Tris-HCl during 24 hours and (right) **ZIF-EC-1** particles incubated with Tris-HCl and DIFP during 24 hours. Experimental conditions: **ZIF-EC-1** (0.005 mmol), Tris-HCl (0.1 M, pH 7.4, 0.5 mL) and DIFP (0.18 M), room temperature.

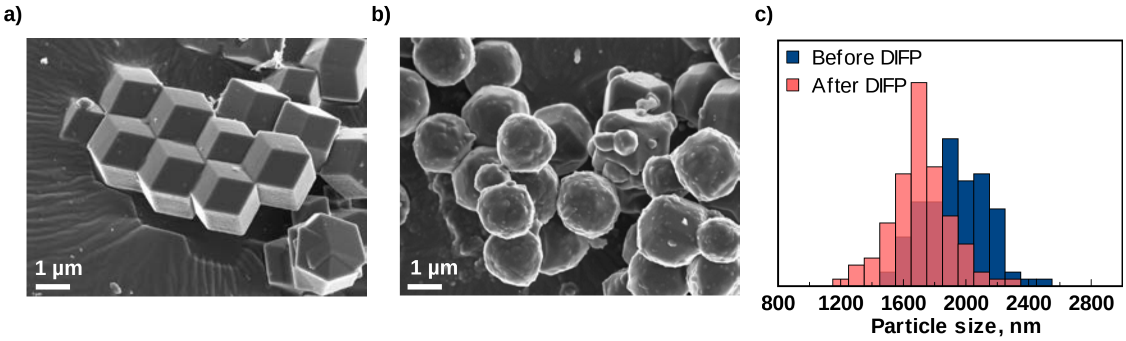

Figure S26. SEM image of (a) pristine **sod ZIF-8_2 µm**, and (b) **sod ZIF-8_2 µm** incubated with Tris-HCl and DIFP for 24 hours. Experimental conditions: **sod ZIF-8_2 µm** (0.015 mmol), Tris-HCl (0.1 M, pH 7.4, 0.5 mL) and DIFP (0.18 M), room temperature. (c) Particle size distribution of **sod ZIF-8_1.2 µm** before (blue bars) and after (blue bars) DIFP treatment

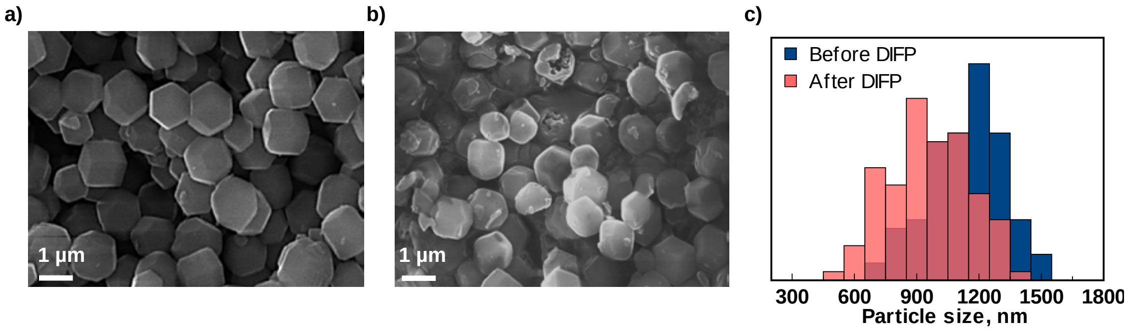

Figure S27. SEM image of (a) pristine **sod ZIF-8_1.2 µm**, and (b) **sod ZIF-8_1.2 µm** incubated with Tris-HCl and DIFP for 24 hours. Experimental conditions: **sod ZIF-8_1.2 µm** (0.015 mmol), Tris-HCl (0.1 M, pH 7.4, 0.5 mL) and DIFP (0.18 M), room temperature. (c) Particle size distribution of **sod ZIF-8_1.2 µm** before (blue bars) and after (blue bars) DIFP treatment.

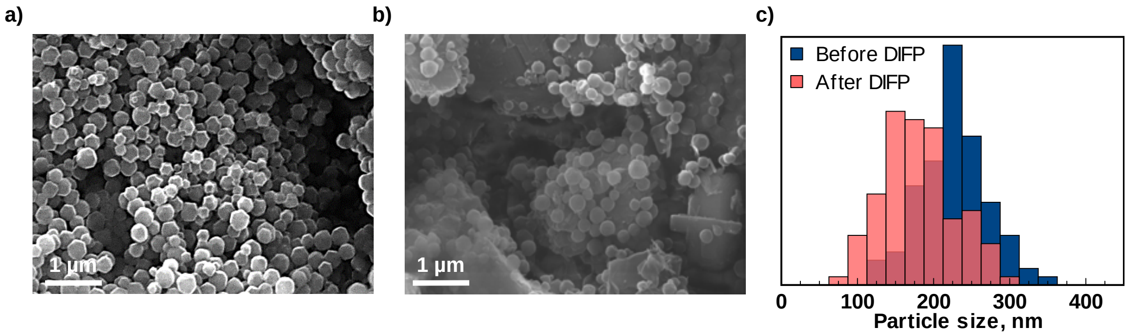

Figure S28. SEM image of (a) pristine sod **ZIF-8_240 nm**, and (b) **sod ZIF-8_240 nm** incubated with Tris-HCl and DIFP for 24 hours. Experimental conditions: **sod ZIF-8_240 nm** (0.015 mmol), Tris-HCl (0.1 M, pH 7.4, 0.5 mL) and DIFP (0.18 M), room temperature. (c) Particle size distribution of **sod ZIF-8_240 nm** before (blue bars) and after (blue bars) DIFP treatment.

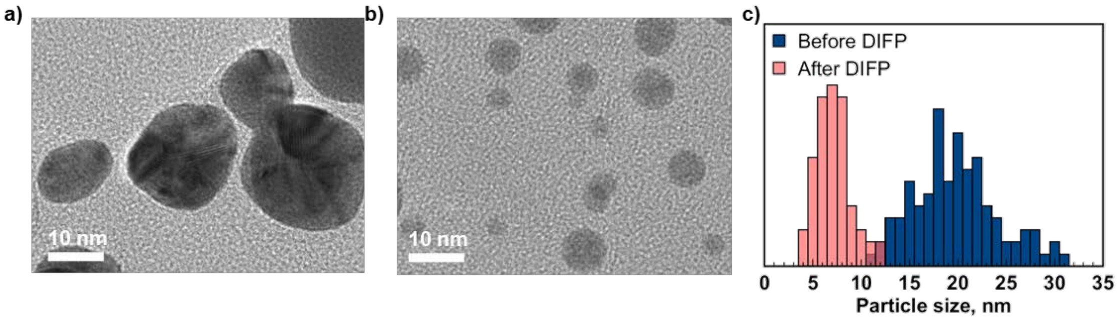

Figure S29. TEM image of (a) pristine sod **ZIF-8_20 nm**, and (b) **sod ZIF-8_20 nm** incubated with Tris-HCl and DIFP for 24 hours. Experimental conditions: **sod ZIF-8_20 nm** (0.015 mmol), Tris-HCl (0.1 M, pH 7.4, 0.5 mL) and DIFP (0.18 M), room temperature. (c) Particle size distribution of **sod ZIF-8_20 nm** before (blue bars) and after (blue bars) DIFP treatment.

S.3.4. Inductively coupled plasm mass spectroscopy (ICP-MS)

Sod ZIF-8_2 μm (0.084 mmol), ZIF-L (0.084 mmol) and ZIF-EC-1 (0.029 mmol) were incubated in Tris-HCl (0.1 M, pH 7.4, 0.5 mL) during 24 h at room temperature. Supernatants of sod ZIF-8, ZIF-L and ZIF-EC-1 were collected after centrifugation and were diluted properly in 2% nitric acid aqueous solution prior to ICP-MS measurements.

Table S5. Zn^{2+} ions released from sod ZIF-8, ZIF-L and ZIF-EC-1 after incubation in Tris-HCl (0.1 M, pH 7.4, 0.5 mL) at room temperature during 24 h expressed as percentage of whole Zn content and Zn^{2+} ion concentration.

	**Zn^{2+} released from ZIF (%)**	**[Zn^{2+}] supernatant (mM)**
**Sod ZIF-8**	2.93	4.93
**ZIF-L**	3.33	5.73
**ZIF-EC-1**	5.27	8.72

S.4. Enzymatic assays.

S.4.1. AChE Reactivation assays.

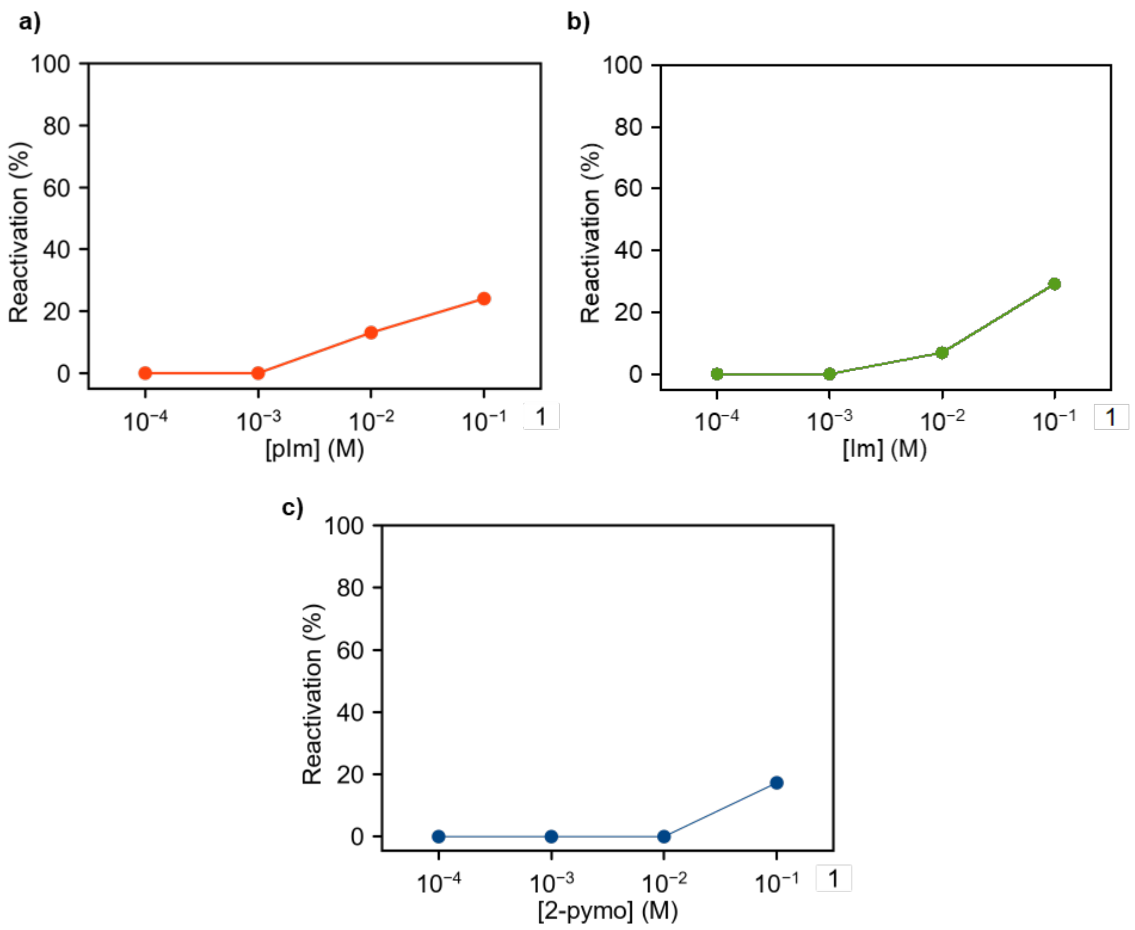

Figure S30. (a) Reactivation profile of DIFP inhibited AChE by (a) free pIm (red curve), (b) free Im (green curve) and (c) free 2-pymo (blue curve).

S.4.2. Detoxification studies.

Table S6. Reactivation of DIFP inhibited-AChE by imidazolate linkers released from ZIFs degradation after 24 h incubation in Tris-HCl (0.1 M, pH 7.4). Detoxifying activity of ZIFs upon exposure to DIFP for 1 h, 3 h and 24 h.

	REACTIVATION	DETOXIFICATION		
	24 h (%)	1 h (%)	3 h (%)	24 h (%)
**ZIF-L**	12.8 ± 3.7	14.2	83.3	100
**ZIF-EC-1**	9.0 ± 0.4	17.3	71.7	100
**Sod ZIF-8_2 µm**	37.1 ± 3.8	7.1	13.3	100
**Sod ZIF-8_20 nm**	53.5 ±8.7	91.3	100	100

The detoxification percentage was calculated according to the following expression:

$$Detoxification\ (\%) = \left(1 - \frac{I_{ZIF}}{I_{INH}}\right) \times 100$$

$I_{ZIF} = AChE\ inhibition\ percentage\ for\ ZIFs\ experiments$

$I_{INH} = AChE\ inhibition\ percentage\ for\ inhibition\ control\ experiments$

S.5. References

[1] J. I. Deneff, K. S. Butler, P. G. Kotula, B. E. Rue, D. F. Sava Gallis, *ACS Appl. Mater. Interfaces* **2021**, *13*, 27295.

[2] M. He, J. Yao, Q. Liu, Z. Zhong, H. Wang, *Dalt. Trans.* **2013**, *42*, 16608.

[3] N. Masciocchi, G. A. Ardizzoia, S. Brenna, F. Castelli, S. Galli, A. Maspero, A. Sironi, *Chem. Commun.* **2003**, *3*, 2018.

[4] J. Cravillon, R. Nayuk, S. Springer, A. Feldhoff, K. Huber, M. Wiebcke, *Chem. Mater.* **2011**, *23*, 2130.

[5] R. Chen, J. Yao, Q. Gu, S. Smeets, C. Baerlocher, H. Gu, D. Zhu, W. Morris, O. M. Yaghi, H. Wang, *Chem. Commun.* **2013**, *49*, 9500.

[6] M. Ge, Y. Wang, F. Carraro, W. Liang, M. Roostaeinia, S. Siahrostami, D. M. Proserpio, C. Doonan, P. Falcaro, H. Zheng, X. Zou, Z. Huang, *Angew. Chem. Int. Ed.* **2021**, *60*, 11391.

[7] N. Masciocchi, G. A. Ardizzoia, G. LaMonica, A. Maspero, A. Sironi, *Eur. J. Inorg. Chem.* **2000**, *2000*, 2507.

[8] M. Pohanka, M. Hrabinova, K. Kuca, J. P. Simonato, *Int. J. Mol. Sci.* **2011**, *12*, 2631.

[9] J. Seixas De Melo, A. P. Moura, M. J. Melo, *J. Phys. Chem. A* **2004**, *108*, 6975.
